# ﻿How many more species are out there? Current taxonomy substantially underestimates the diversity of bent-toed geckos (Gekkonidae, *Cyrtodactylus*) in Laos and Vietnam

**DOI:** 10.3897/zookeys.1097.78127

**Published:** 2022-04-26

**Authors:** Hanh Thi Ngo, Quyen Hanh Do, Cuong The Pham, Vinh Quang Luu, L. Lee Grismer, Thomas Ziegler, Van Thi Hong Nguyen, Truong Quang Nguyen, Minh Duc Le

**Affiliations:** 1 Central Institute for Natural Resources and Environmental Studies, Vietnam National University, 19 Le Thanh Tong, Hanoi, Vietnam; 2 Faculty of Biology, Hanoi University of Science, 334 Nguyen Trai Road, Thanh Xuan, Hanoi, Vietnam; 3 AG Zoologischer Garten Köln, Riehler Strasse 173, D-50735 Cologne, Germany; 4 Institute of Zoology, University of Cologne, Zülpicher Strasse 47b, D-50674 Cologne, Germany; 5 Faculty of Environmental Sciences, Hanoi University of Science, 334 Nguyen Trai Road, Thanh Xuan, Hanoi, Vietnam; 6 Institute of Ecology and Biological Resources and Graduate, University of Science and Technology, Vietnam Academy of Science and Technology, 18 Hoang Quoc Viet, Cau Giay, Hanoi, Vietnam; 7 Faculty of Forest Resources and Environmental Management, Vietnam National University of Forestry, Xuan Mai, Chuong My, Hanoi, Vietnam; 8 Department of Herpetology, San Diego Natural History Museum, PO Box 121390, San Diego, California, 92112, USA; 9 Herpetology Laboratory, Department of Biology, La Sierra University, 4500 Riverwalk Parkway, Riverside, California 92505, USA; 10 Department of Herpetology, American Museum of Natural History, Central Park West at 79th Street, New York, New York 10024

**Keywords:** COI, conservation, Gekkonidae, integrative taxonomy, Southeast Asia, synonymy

## Abstract

*Cyrtodactylus* is the most diverse genus of the family Gekkonidae and the world’s third largest vertebrate genus. The number of species has increased more than fourfold over the last two decades. Indochina, especially Vietnam and Laos, has witnessed a surge in new species discoveries over the last three decades. The species number reported from Laos and Vietnam has remarkably increased from five in 1997 to 71 species in 2021. However, within the genus, several taxonomic issues have not yet been fully resolved. Based on recently collected samples from Laos and Vietnam, we conducted a comprehensive molecular review of *Cyrtodactylus* occurring in Laos and Vietnam. Our molecular analysis with support from morphological comparisons showed that *C.thuongae* is a junior synonym of *C.dati* and *C.rufford* is a junior synonym of *C.lomyenensis*. In total, 68 described species distributed in Laos and Vietnam are undisputed with strong support from both molecular and morphological evidence. On the other hand, the molecular analyses revealed that there are at least seven undescribed species in Vietnam and Laos, one in the *C.angularis* group, one in the *C.chauquangensis*, and five in the *C.irregularis* group. This number will likely increase significantly, as previous work suggested that the *C.angularis* and *C.irregularis* groups harbor three and six unnamed lineages, respectively. Based on survey gaps identified in our study, it is clear that additional new species will be discovered in poorly studied regions of central Vietnam and northern and southern Laos. As many species in the genus are facing high extinction risks, several undescribed populations might already be severely threatened by human activities in both countries. Therefore, urgent taxonomic research is needed before conservation assessments of newly discovered taxa can be undertaken to protect them from anthropogenic threats.

## ﻿Introduction

The bent-toed geckos of the genus *Cyrtodactylus* comprise the most diverse genus of the Gekkonidae with at least 330 nominal species (Uetz et al. 2021). They have a broad distribution extending from tropical South Asia, Indochina, the Philippines, and the Indo-Australian Archipelago to the Solomon Islands (Grismer et al. 2021a, b). Species in the genus can adapt to different habitat types, including limestone karst, granitic montane forests, and lowland evergreen forest. Interestingly, several species have been observed in sympatry, for example, in Phong Nha – Ke Bang National Park in Vietnam (*C.cryptus* Heidrich, Rösler, Vu, Böhme & Ziegler, 2007; *C.phongnhakebangensis* Ziegler, Rösler, Herrmann & Vu, 2003; *C.roesleri* Ziegler, Nazarov, Orlov, Nguyen, Vu, Dang, Dinh and Smith, 2010) and Ba Den Mountain in Vietnam (*C.badenensis* Nguyen, Orlov & Darevsky, 2006; *C.nigriocularis* Nguyen, Orlov & Darevsky; *C.thuongae* Phung, van Schingen, Ziegler & Nguyen, 2006) (Ziegler et al. 2003, 2010, 2013; Nguyen et al. 2006; Heidrich et al. 2007; Phung et al. 2014). Additionally, many new species of *Cyrtodactylus* have been described over the last ten years. *Cyrtodactylus* is therefore recognized as an ideal group for taxonomic, biogeographic, and ecological research as well as a model group for lizard evolution (Grismer et al. 2021a, b; 2022).

Indochina, including Cambodia, Laos, and Vietnam, has long been recognized as a region of global importance in terms of biodiversity richness (Myers et al. 2000). Laos and Vietnam have also been a hotspot of new *Cyrtodactylus* discoveries. From 1997 to present, 66 new species of *Cyrtodactylus* have been described, making it a total of 71 recognized species (Uetz et al. 2021). Remarkably, many cryptic species have recently been described based on either comparison with newly acquired collections or implementation of an integrative approach, i.e., combining evidence from morphological and molecular data. For example, *C.phongnhakebangensis* was split into two species, namely *C.phongnhakebangensis* and *C.roesleri* in 2010 (Ziegler et al. 2010), which were found to occupy different ecological niches (Loos et al. 2012), and *C.irregularis* Smith, 1921 was broken up into multiple species (Nazarov et al. 2008; Geissler et al. 2009; Grismer et al. 2021a, b). On the other hand, several species have been synonymized. *C.paradoxus* Darevsky & Szczerbak, 1997 was shown to be a junior synonym of *C.condorensis* Smith, 1921 and *C.thochuensis* Ngo & Grismer, 2012 was recommended as a junior synonym of *C.leegrismeri* Chan & Norhayati, 2010 (Orlov et al. 2007; Grismer et al. 2015). In addition, *C.thuongae* was synonymized with *C.dati* Ngo, 2013 based on molecular evidence (Ngo et al. 2017).

In Laos, most new species described in recent years belong to the *Cyrtodactylusangularis* group, which contains at least 16 species recorded in the country. This karst-adapted clade occurs in central Laos and north-central Vietnam (Grismer et al. 2021a). Another five recently discovered species are members of the *C.chauquangensis* group, which occupies northern Laos and northwestern and north-central Vietnam. Two remaining groups include *C.brevipalmatus* and *C.irregularis*. While it is still unclear what species of the former group are present in Laos, the latter likely consists of three species in Laos, *C.buchardi* David, Teynié, Ohler, 2004; *C.cryptus*, and *C.pseudoquadrivirgatus* Rösler, Nguyen, Vu, Ngo & Ziegler, 2008 (David et al. 2004; Rösler et al. 2008; Pauwels et al. 2018; Schneider et al. 2020; Grismer et al. 2021a)

As new species of the genus have been consistently described at a rapid rate, there is a need to review the taxonomic progress and identify areas where future research should focus. Although there have been some attempts to assess the diversity of the group in Vietnam and Laos using molecular data (Nguyen et al. 2015, 2017; Brennan et al. 2017; Ngo et al. 2017), the studies either did not include a thorough taxonomic sampling of species in both countries (Brennan et al. 2017; Grismer et al. 2021a, b) or only focused on Vietnamese or Lao taxa (Nguyen et al. 2017; Ngo et al. 2017; Schneider et al. 2020). To better understand the outstanding taxonomic issues and accurately evaluate the taxonomic diversity of the group, we analyzed 68 of 71 described species and several undescribed populations from across the range of this group in Laos and Vietnam. To determine the distribution ranges of the taxa, we incorporated as many samples as possible from different localities and samples from newly discovered and undescribed populations from previous studies. In some cases where the molecular evidence was equivocal, morphological comparisons were employed to address the pending taxonomic issues.

## ﻿Materials and methods

### ﻿Sampling

Field work was conducted between 2009 and 2018 in Laos and Vietnam. Specimens were euthanized with ethyl acetate, fixed in approximately 85% ethanol, then transferred to 70% ethanol for permanent storage. Specimens were subsequently deposited in the collections of the Institute of Ecology and Biological Resources (**IEBR**), Vietnam Academy of Science and Technology, Hanoi, Vietnam; the Vietnam National Museum of Nature (**VNMN**), Hanoi, Vietnam; the Vietnam National University of Forestry (**VNUF**), Hanoi, Vietnam; the National University of Laos (**NUOL**), Laos; and the Zoological Research Museum Alexander Koenig (**ZFMK**), Bonn, Germany.

### ﻿Morphological analysis

Main morphological characters were rechecked: Measurements were taken with a digital caliper to the nearest 0.1 mm. Abbreviations are as follows: snout-vent length (**SVL**, from tip of snout to anterior margin of cloaca); tail length (**TaL**, from posterior margin of cloaca to tip of tail).

Scale counts were taken using stereo microscopes (Leica S6E, Keyence VHX-500F): ventral scales in longitudinal rows at midbody (**V**) counted transversely across the center of the abdomen from one ventrolateral fold to the other; dorsal tubercle rows (**DTR**) counted transversely across the center of the dorsum from one ventrolateral fold to the other; supralabials (**SL**) and infralabials (**IL**) counted from the first labial scale to the corner of mouth; enlarged femoral scales (**EFS**); femoral pores (**FP**); precloacal pores (**PP**) or the total number of femoral pores and precloacal pores (i.e. the contiguous rows of femoral and precloacal scales bearing pores combined as a single meristic character referred to as the femoroprecloacal pores); number of subdigital lamellae on the fourth finger (**LD4**) and number of subdigital lamellae on the fourth toe (**LT4**) counted from the base of the first phalanx to the claw.

### ﻿Molecular data and phylogenetic analysis.

Most described taxa of the genus *Cyrtodactylus* in Laos and Vietnam, except for *C.buchardi*, *C.raglai*, and *C.septimontium* were included in the study. In addition, samples of the species from different localities were sequenced to determine their distribution range. In total, 84 new samples from 26 provinces were incorporated (Suppl. material 1: Table S1). The tissue samples of muscle, liver or tail tissue was preserved separately in 70% ethanol. The mitochondrial DNA cytochrome c oxidase subunit I (COI) gene was selected as the markers have been widely used in previous studies and for some geographic populations, only COI data were available (Nguyen et al. 2015, 2017; Luu et al. 2016a; Brennan et al. 2017; Ngo et al. 2017). In several cases, where comparative data for COI were not available, the mitochondrial gene NADH dehydrogenase subunit 2 (ND2) was generated for specimens under consideration. In addition, we obtained 90 sequences of the mitochondrial COI for the ingroup taxa and one outgroup species, *Hemidactylusfrenatus*, from GenBank (Wood et al. 2012).

Total genomic DNA was extracted using DNeasy Blood and Tissue Kit (Qiagen, Germany), following protocols by the manufacturer’s instructions. PCR was performed using HotStar Taq Mastermix (Qiagen, Germany) to amplify approximately 657 bp fragment of the mitochondrial gene COI and approximately 1200 bp fragment of the mitochondrial gene ND2. We used two primer pairs for PCR with VF1d (5’–TTCTCAACCAACCACAARGAYATYGG-3’), VR1d (5’– TAGACTTCTGGGTGGCCRAARAAYCA–3’) (Ivanova et al. 2006) for generating the COI and MetF1 (5’– AAGCTTTCGGGCCCATACC–3’), COIR1 (5’– AGRGTGCCAATGTCTTTGTGRTT–3’) (Macey et al. 1997 for the ND2. The PCR volume consisted of 21 µl (2 µl each primer, 5 µl water, 10 µl of Taq mastermix and 1 – 2 µl of DNA depending on the quality of DNA in the final extraction solution). The reactions were performed at 95 °C for 15 min, followed by 35 cycles of 30 s at 95 °C, 45 s at 48 °C, and 60 s at 72 °C with a final elongation step of 6 min at 72 °C. A negative and positive control was used for every DNA extraction and PCR reactions. PCR products were visualized using electrophoresis through a 1% agarose gel, marker 1 kb, 1X TBE and stained with ethidium bromide and photographed under UV light. Successful amplifications were purified using GeneJet PCR Purification Kit (ThermoFisher Scientific, Lithuania). Cleaned PCR products was sent to 1^st^ Base (Malaysia) for sequencing.

Newly generated sequences were checked by eye using Sequencher v5.4 (Gene Codes Corp, Ann Arbor, MI, USA), aligned by ClustalX v2.1 (Thompson et al. 1997) with default setting. The data were then analyzed using Bayesian inference (BI) as implemented in MrBayes v3.1.2 (Ronquist et al. 2012) and maximum likelihood (ML) analysis using IQ-TREE v.1.6.7.1 (Nguyen et al. 2015) with a single molecular model and 10,000 ultrafast bootstrap (UFBP) replications. For BI, the analysis was conducted with a random starting tree and run for 1 × 10^7^ generations. Four Markov chains, one cold and three heated (utilizing default heating values), were sampled every 1000 generations. Log likelihood scores of sample points were plotted against generation time to detect stationarity of the Markov chains. The burn-in value was set to 59 in the BI analysis, as –lnL scores reached stationarity after 59,000 generations in both runs. The optimal model, GTR+I+G, for nucleotide evolution was set to BI and ML analysis as selected by jModeltest v2.1.4 (Darriba et al. 2012). Nodal support was evaluated using UFBP as estimated in IQ-TREE v1.6.7.1 and posterior probability (PP) in MrBayes v3.2. UFBP and PP ≥ 95% are regarded as strong support for a clade (Ronquist et al. 2012; Nguyen et al. 2015). Uncorrected pairwise distance (*p*) was calculated in PAUP*v4.0b10 (Swofford 2001).

## ﻿Results

### ﻿Phylogenetic relationships

**Figure 1.**
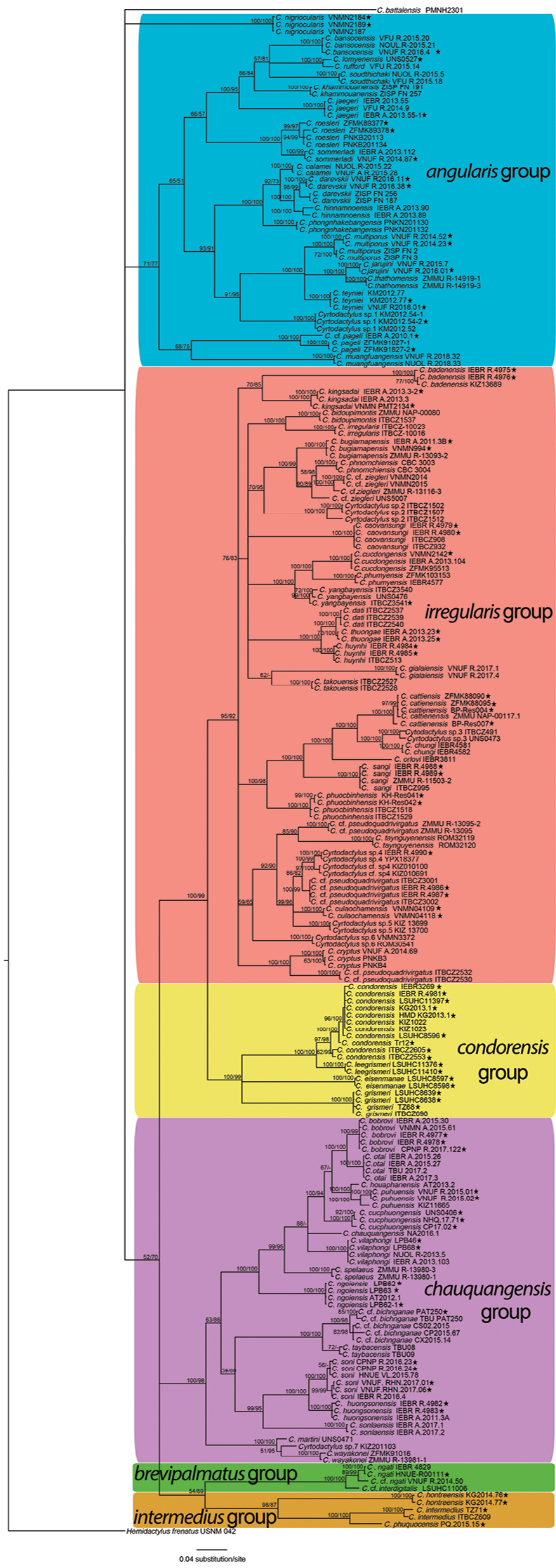
Bayesian cladogram based on 657 bp of the partial COI gene. Numbers above branches are Bayesian posterior probabilities and ultrafast bootstrap values of ML analysis, respectively. ★ = new sequences used in the phylogenetic analyses.

We successfully sequenced a fragment of the COI gene for 90 samples and ND2 gene for four samples. The final concatenated matrix consisted of 216 terminals, including 90 from this study, 126 from previous studies, including one outgroup, *Hemidactylusfrenatus*, following Wood et al. (2012). Both BI and ML analyses based on a total of 657 aligned characters with no gaps and using a single model of molecular evolution produced very similar topologies (Fig. 1). All species groups were generally recovered with strong support values from both analyses, except for the *C.irregularis* group, which was corroborated only by the BI analysis (Fig. 1). The results show that *Cyrtodactylus* species in Laos and Vietnam fall into six species groups, namely *C.angularis*, *C.brevipalmatus*, *C.chauquangensis*, *C.condorensis*, *C.intermedius*, and *C.irregularis*. While Vietnam harbors members of all groups, Lao species mostly belong to the two karst-dwelling *C.angularis* and *C.chauquangensis* groups (Grismer et al. 2021a, b). Two other groups contain one taxon each from Laos, including C.cf.cryptus of the *C.irregularis* group (samples of *C.buchardi* and *C.pseudoquadrivirgatus* from Laos not included in the analysis) and C.cf.ngati of the *C.brevipalmatus* group. Two remaining mostly insular groups, the *C.condorensis* and *C.intermedius* groups, do not have any species from Laos (Fig. 1, Suppl. material 1: Table S1).

The main difference between this and previous studies is that the *Cyrtodactylusangularis* group was rendered paraphyletic. Nguyen et al. (2015) and Ngo et al. (2017) used the same COI region and confirmed that the monophyly of the group is significantly supported in the BI analysis, while its monophyletic relationship based on 1474 base pairs of the ND2 gene received perfect statistical values from both BI and ML phylogenetic estimates (Grismer et al. 2021a). All other species groups were recovered with strong support values from both analyses, except for the *C.irregularis* group, which was corroborated only by the BI analysis but received perfect statistical support in both analysis in Grismer et al. (2021a).

In the *Cyrtodactylusangularis* group, almost all species are well defined and supported by both analyses. According to our tree, the group contains 19 known species and one undescribed taxon (*Cyrtodactylus* sp. 1) in both countries. There are only three species that have notable genetic sub-structuring, i.e., *C.darevskii*, *C.multiporus*, and *C.pageli*, and samples from genetically distinct populations are labeled as cf. (Fig. 1). Another population from Khammouane Province, Laos with three representative samples, KM2012.52, KM2012.54–1, and KM2012.54–2, is clearly differentiated from other species and likely forms a new species. The former two samples were incorrectly assigned to *C.lomyenensis* in previous studies (Nazarov et al. 2014, Schneider et al. 2014). It is marked as *Cyrtodactylus* sp. 1 on the tree (Fig. 1). The highest pairwise genetic divergence between species of the *Cyrtodactylusangularis* group is 24.04% based on the fragment of COI gene (Suppl. material 2: Table S2). The lowest pairwise genetic divergence between species that are not morphologically well differentiated (SVL, LT4, LD4, Infralabials, EFS, color pattern of dorsum, and enlarged subcaudals specifications, see Tables 2, 4) is 2.44% (*C.rufford* and *C.lomyenensis*). The lowest divergence between species that are morphologically distinct is 4.57% (*C.jarujini* and *C.thathomensis*).

Members of the *Cyrtodactylusbrevipalmatus* group, recently discovered in Vietnam (Le et al. 2021), are present in both countries. The genetic divergence between the Vietnamese and Lao populations of *C.ngati* is 2.13% and they are ~ 3.81–4.41% separated from C.cf.interdigitalis from Thailand. This group contains the lowest number of taxa (Fig. 1, Suppl. material 1: Table S1, Suppl. material 6: Table S6). The *C.chauquangensis* group is also distributed in both countries with 16 described species and one undescribed form, *Cyrtodactylus* sp. 7, from Vietnam. Samples of purported *C.bichnganae* are all labeled as cf. because genetic sequences of the true *C.bichnganae* have not been made available (see Pham et al. 2019). The second and third smallest groups, the *C.intermedius* and *C.condorensis* groups, have three and four species, respectively. All of the species occur in Vietnam and mostly inhabit offshore islands in the southern part of the country.

The largest group, the *Cyrtodactylusirregularis* group, consists of more than 30 species with at least five undescribed forms, *Cyrtodactylus* sp. 2 – *Cyrtodactylus* sp. 6. The highest and lowest pairwise genetic divergence that exists between species of the *C.irregularis* group are 21.41% and 0.74% (Suppl. material 3: Table S3). In addition, C.cf.pseudoquadrivirgatus is recovered in three distinct places of the tree, one close to *C.taynguyenensis*, one embedded within a clade comprising *Cyrtodactylus* sp. 4, *Cyrtodactylus* sp. 5, *C.culaochamensis*, and the other was recovered as a sister taxon to *C.cryptus*. Finally, *C.dati* and *C.thuongae* are not highly divergent in terms of pairwise genetic distance and only separated by 0.74% (Table 3, Suppl. material 3: Table S3).

The *Cyrtodactyluscondorensis* group is composed of four well defined species with pairwise divergences of 5.48 – 18.05% (Suppl. material 4: Table S4). The *Cyrtodactyluschauquangensis* group is composed of 17 species which have remained stable in many analyses of phylogenetic relationships. The genetic divergences between the members of the group are ~ 3.81–19.54% (Suppl. material 5: Table S5).

### ﻿Taxonomic issues

Our results based on a fragment of the mitochondrial gene COI show that the lineage containing *Cyrtodactylusthuongae* with the holotype and paratype from Tay Ninh Province and *C.dati* from the Lam Dong, Binh Phuoc populations is divided into two sub-lineages. However, the PP value is insignificant (Fig. 1). These populations are separated by no greater than 1% in sequence divergence using COI data (Table 3). In addition, genetic distances between the holotype of *C.dati* (UNS 0543) and the holotype and paratype of *C.thuongae* (IEBR A.2013.23, IEBR A.2013.25) are 2.21% and 1.85%, respectively, based on 951 bp of a fragment of the mitochondrial ND2 gene. At the time of the latter species’ description, molecular data for both species were not available for comparison. Instead, *C.thuongae* was described on the basis of its highly variable morphology (Table 1). Morphological examination of specimens of *C.thuongae* and published data of *C.dati* show that morphologically they do not have a high level of distinction (size, number of ventral scales, infralabials, EFS, FP, LT4, enlarged subcaudals, color pattern of dorsum. The only differences between the two species are: DTR: 17 or 18 in *C.thuongae* vs. 20–22 in *C.dati*, SL: 7–9 in *C.thuongae* vs. 10–12 in *C.dati* and 0 or 1 pitted scales in males in *C.thuongae* vs. five or six in *C.dati*.

**Table 1.** Morphological characters of *Cyrtodactylusdati*, *C.huynhi*, *C.thuongae*.

	*C.dati*	*C.thuongae*	*C.huynhi* without UNS 0327	*C.huynhi* UNS 0327 paratype, M
Article	Ngo 2013	Phung et al. 2014	Ngo et al. 2008	Ngo and Bauer 2008
Locality	Binh Phuoc	Tay Ninh	Dong Nai	Dong Nai
SVL	max 70.1	57.3 – 77.6	67.2 – 79.8	54.8
TaL	max 57.3	max 78.1	61.5 – 78.6	29.1
V	42 – 48	29 – 44	43 – 46	44
DTR	20 – 22	17 – 18	16 – 18	18
SL	10 – 12	7 – 9	?	?
IL	8 – 10	7 – 10	?	?
EFS	4 – 7	2 – 5	3 – 5	/
FP	3 – 4 each	0 – 3 (pitted scales)	3 – 8	4+4
PP in males	5 – 6	0 – 1 (pitted scales)	7 – 9	9
PP in females	?	0	0 – 8 (pitted scales)	?
LD4	?	14 – 17	14 – 17	15
LT4	18 – 19	14 – 20	17 – 21	17
Color pattern of dorsum	blotched	blotched	banded	banded
Enlarged subcaudals	absent	absent	absent	absent

Much the same is true for *C.lomyenensis* from Khammouan (paratype – UNS0527) and *C.rufford* also from Khammouan (holotype – VFU R.2015.14). Genetic divergence between the two species is less than 2.44% based on a fragment of mitochondrial gene COI. According to our morphological examinations between specimens of *C.rufford* and published data of *C.lomyenensis*, *C.rufford* differs from *C.lomyenensis* by having fewer ventral scale rows (27–29 vs. 35 or 36), fewer supralabials (10–12 vs. 13 or 14), and more femoral and precloacal pores in males (42 or 43 vs. 39 or 40) (Table 2). The differences are quite small, except for the ventral scale row. In addition, *C.rufford* is similar to *C.lomyenensis* in size and coloration: head dorsum yellowish with irregular brown blotches, dorsal pattern with transverse bands, rings on original tail with dark brown transversal bands wider than light brown spaces, and median row of enlarged subcaudal scales. In addition, genetic distance between the paratype of *C.lomyenensis* (UNS0527) and holotype of *C.rufford* (VFU R.2015.14) is 0.21% based on 413 bp of a fragment of the mitochondrial ND2 gene.

**Table 2.** Morphological characters of *Cyrtodactylusrufford* and *C.lomyenensis*.

	*C.lomyenensis*	*C.rufford*
Article	Ngo and Pauwels 2010	Luu et al. 2016b
Locality	Khammouan Province	Khammouan Province
SVL	max 71.2	max 72.5
TaL	max 86.1 (Reg)	max 96.8
V	35 – 36	27 – 29
DTR	20 – 24	14 – 16
SL	13 – 14	11 – 12
IL	11	9 – 11
EFS	17 – 18	17 – 18
Total of FP and PP in males	39 – 40	42 – 43
LD4	16 – 19	19 – 20
LT4	19 – 23	18 – 19
Color pattern of dorsum	four narrow yellowish-cream transversal bands	three or four light transverse bands
Enlarged subcaudals	medially enlarged	medially enlarged

## ﻿Discussion

In general, the phylogenetic relationships supported by this study are similar to those corroborated by previous studies using the same genetic marker (Nguyen et al. 2013; Luu et al. 2016a; Brennan et al. 2017; Ngo et al. 2017). However, some outstanding issues remain unresolved. Specifically, this study shows that *C.badenensis* is a member of the *C.irregularis* group, although Grismer et al. (2021a) based on ND2 suggested that the species belongs to *C.condorensis* or an independent lineage. The *C.irregularis* group contains several taxonomically unconfirmed populations. For example, C.cf.ziegleri revealed to be at least two taxa, while the phylogenetic placement of the true *C.ziegleri*, similarly to the previously reported cases in *C.bichnganae* in the *C.chauquangensis* group, has not been clarified in previous studies. Our study also confirms that although COI is a good marker for DNA barcoding it is limited by its length and lacks characters to resolve deeper nodes.

**Table 3.** Uncorrected (“p”) distance matrix showing percentage genetic divergence (COI) between *Cyrtodactylusdati*, *C.thuongae*, and closely related species. Numbers in bold are the lowest percentages.

Species	1	2	3	4	5	6	7	8	9	10	11	12
1. *C.bidoupimontis*HQ967215	–											
2. *C.bugiamapensis*IEBR A.2011.3B	13.24	–										
3. *C.caovansungi* NT.2016.2	15.07	14.0	–									
4. *C.cucdongensis*VNMN PMT 2142	13.70	14.16	15.68	–								
5. *C.cryptus*KX064038	14.31	15.53	15.07	14.92	–							
6. *C.dati*KF929508	14.71	15.05	17.07	17.04	16.78	–						
7. *C.huynhi*KF169948	14.18	15.24	16.71	16.68	16.77	**4.18**	–					
8. *C.irregularis*KP199951	8.86	15.13	16.96	14.05	14.97	14.71	14.89	–				
9. *C.takouensis*KF929533	13.26	11.98	13.45	12.32	15.31	13.64	12.36	13.42	–			
10. *C.thuongae*IEBR A.2013.23	14.61	14.61	16.44	16.44	15.53	**0.74**	**3.83**	14.52	13.46	–		
11. *C.yangbayensis* ITBCZ 3540	12.79	12.33	15.22	7.92	15.22	15.22	14.31	12.83	11.60	14.46	–	
12. *C.ziegleri*HQ967210	14.41	7.36	14.41	15.34	15.17	15.42	15.07	15.51	12.59	15.02	13.15	–

*Cyrtodactylus* has a complex taxonomic history and at least two species have been synonymized before this study. Grismer et al. (2015) examined the taxonomy of *C.condorensis* and *C.intermedius* complex using 100 samples from 30 localities and their analyses based on the mitochondrial ND2 suggested that *C.paradoxus* is a junior synonym of *C.condorensis* and *C.thochuensis* is a junior synonym of *C.leegrismeri*. Based on the similarities of morphological and molecular data, we consider *C.thuongae* Phung, van Schingen, Ziegler & Nguyen, 2014 a junior synonym of *C.dati* Ngo, 2013 and place *C.rufford* Luu, Calame, Nguyen, Le, Bonkowski & Ziegler, 2016b in the synonymy of *C.lomyenensis* Ngo & Pauwels, 2010. With two more species synonymized in this study, *Cyrtodactylus* from Vietnam and Laos currently consists of 69 valid species, including 47 from Vietnam and 22 from Laos (of these, *C.cryptus* and *C.roesleri* are known from both countries). Independent of morphological evidence, our molecular phylogenetic results confirm that other lineages represent undisputed species.

**Table 4.** Uncorrected (“p”) distance matrix showing percentage genetic divergence (COI) between *Cyrtodactyluslomyenensis*, *C.rufford* and closely related species. Number in bold is the lowest percentage.

Species	1	2	3	4	5	6	7	8
1. *C.bansocensis*KU175573	–							
2. *C.jaegeri*KT004364	15.09	–						
3. *C.khammouanensis*HM888469	12.04	14.92	–					
4. *C.lomyenensis* UNS0527	11.58	15.22	11.72	–				
5. *C.sommerladi*KJ817437	15.55	15.83	16.44	15.98	–			
6. *C.soudthichaki*KX077904	12.65	14.16	13.55	14.00	15.55	–		
7. *C.roesleri*KF929531	15.97	15.27	16.39	16.13	6.22	15.11	–	
8. *C.rufford*KU175572	11.43	14.61	11.87	**2.44**	17.20	14.46	16.28	–

The number of *Cyrtodactylus* species within six identified species groups will likely change as new discoveries continue to be made at a rapid rate. At least seven unnamed lineages are confirmed by our study, one in the *C.angularis* group, one in the *C.chauquangensis* group, and five others in the *C.irregularis* group. In addition, several species complexes, such as *C.pseudoquadrivirgatus* and *C.ziegleri*, warrant further taxonomic clarification and future studies will probably reveal that some of the lineages within the complexes turn out to be new taxa. Of these, *C.pseudoquadrivirgatus* is most problematic because it was described using the type series from a wide distribution while at the moment, many members of the genus *Cyrtodactylus* are known for their notable site-restricted endemism. It is recommended that the species definition be redefined to the holotype of *C.pseudoquadrivirgatus* from A Luoi in Thua Thien Hue Province (including voucher/field numbers ITBCZ3001, ITBCZ3002, AL.2017.125, AL.2017.126), or to topotypic specimens, viz. the series in case they can be clearly proven to represent that taxon.

**Figure 2.**
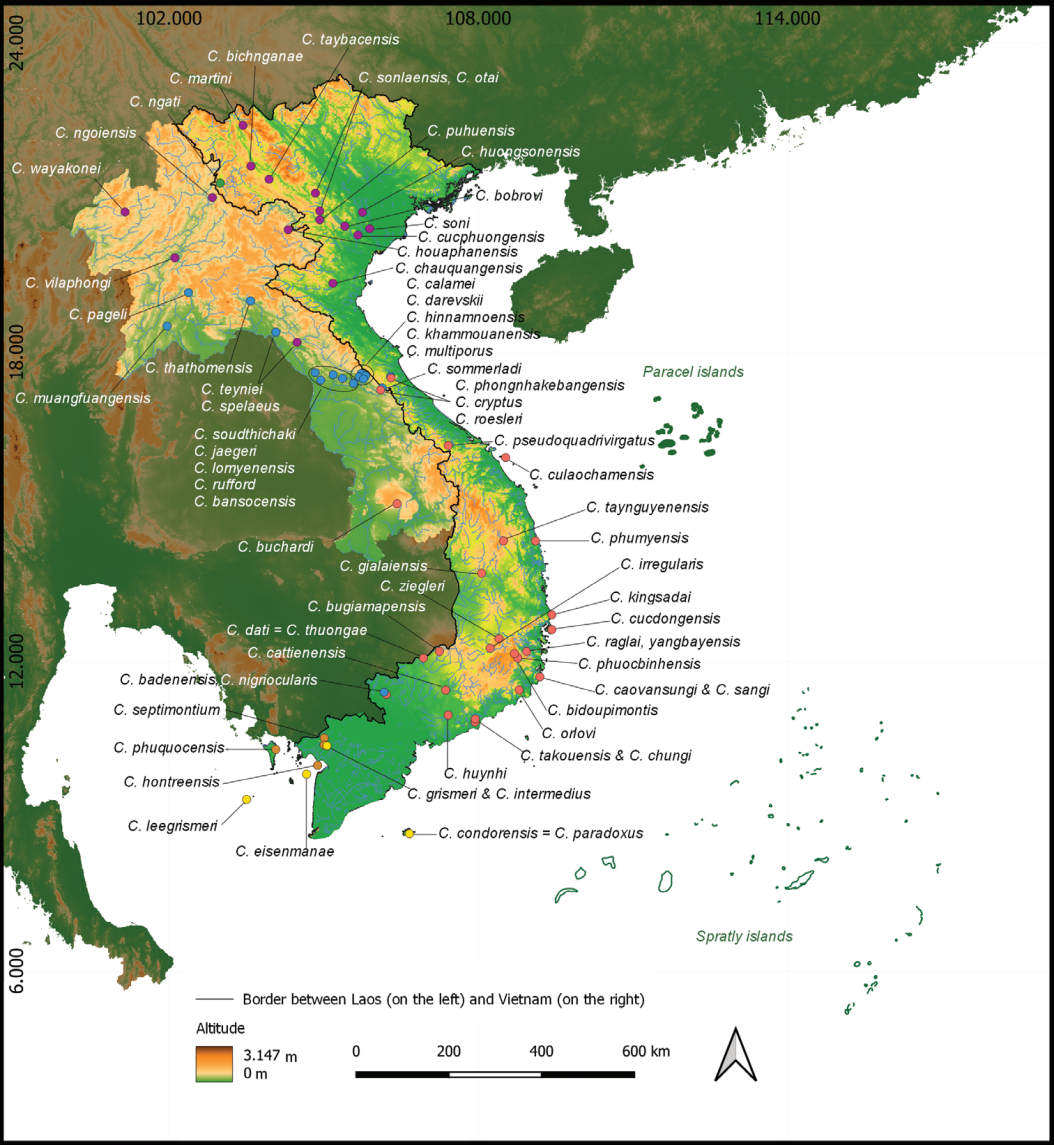
Type localities of all *Cyrtodactylus* taxa occurring in Laos and Vietnam (the altitude data based on GADM database of Global Administrative Areas, 2021).

Grismer et al. (2021a) also suggest that several other unnamed lineages are present in Vietnam and Laos. The *Cyrtodactylusangularis* group comprises three undescribed taxa from Laos, while the *C.irregularis* group includes six. The results of this and our study clearly demonstrate that the *C.irregularis* group is the most speciose within the genus, but there exist many more cryptic species that have not been formally described thus far, in particular from southern Vietnam, a hotspot of this group. It is also noted that several areas in Vietnam and Laos are still poorly studied. More surveys in the areas, in particular the karst region in the northern Annamites, the southern Annamites, and the Central Highlands in Vietnam and northern karst region in northern Laos and the lowland area in southern Laos, will certainly yield more new taxa for science (Fig. 2).

According to the IUCN Red List, several species of this genus in Laos and Vietnam are facing exceedingly high extinction risks, including four species listed as Critically Endangered, three Endangered, and eight Vulnerable (IUCN Red List 2021). The recently described *Cyrtodactylusgialaiensis* is only known from a single locality with a distribution range of less than 10 km^2^ and a population of fewer than 50 individuals (Luu et al. 2017, 2020). However, a majority of *Cyrtodactylus* still need to be carefully evaluated and it is likely that additional assessment will result in a higher number of species to be listed in the IUCN Red List in the future. Taxonomic uncertainty is also hindering conservation efforts, as some undescribed populations might already be critically threatened by human activities in both countries. Urgent research is therefore needed to resolve pending taxonomic issues before conservation assessments for the taxa can be undertaken.

Information on biogeographic ranges of six *Cyrtodactylus* groups occurring in Laos and Vietnam was detailed in Grismer et al. (2021a). According to geographic distribution of our newly collected samples, several species have broader ranges than previously documented. For example, while the occurrences of *C.cattienensis* were reported for the first time from Dong Nai and Ba Ria-Vung Tau provinces, we herein record it from Dong Phu District, Binh Phuoc Province. Distribution of *C.cucdongensis* is extended to Dak Nong Province, *C.huongsonensis* to Lac Thuy District, Hoa Binh Province, *C.kingsadai* to Khanh Hoa and Dak Nong provinces, and *C.phuocbinhensis* to Khanh Hoa Province. With more sampling of the members of the genus in Laos and Vietnam, our knowledge of its taxonomy, distribution, and conservation in the two countries will be improved in the future.

## Supplementary materials

Supplementary material 1 Table S1 Explanation note Samples used in this study. https://binary.pensoft.net/file/677231This dataset is made available under the Open Database License (http://opendatacommons.org/licenses/odbl/1.0/). The Open Database License (ODbL) is a license agreement intended to allow users to freely share, modify, and use this Dataset while maintaining this same freedom for others, provided that the original source and author(s) are credited.Hanh Thi Ngo, Quyen Hanh Do, Cuong The Pham, Vinh Quang Luu, L. Lee Grismer, Thomas Ziegler, Van Thi Hong Nguyen, Truong Quang Nguyen, Minh Duc LeData type: Docx file.

Supplementary material 2 Table S2 Data type Docx file. Explanation note Uncorrected (“p”) distance matrix showing percentage genetic divergence (COI) (highlighted in bold are the lowest and highest percentage) between species in the *Cyrtodactylusangularis* group. https://binary.pensoft.net/file/677232This dataset is made available under the Open Database License (http://opendatacommons.org/licenses/odbl/1.0/). The Open Database License (ODbL) is a license agreement intended to allow users to freely share, modify, and use this Dataset while maintaining this same freedom for others, provided that the original source and author(s) are credited.Hanh Thi Ngo, Quyen Hanh Do, Cuong The Pham, Vinh Quang Luu, L. Lee Grismer, Thomas Ziegler, Van Thi Hong Nguyen, Truong Quang Nguyen, Minh Duc Le

Supplementary material 3 Table S3 Data type Docx file. Explanation note Uncorrected (“p”) distance matrix showing percentage genetic divergence (COI) (highlighted in bold are the lowest and highest percentage) between species in the *Cyrtodactylusirregularis* group. https://binary.pensoft.net/file/677233This dataset is made available under the Open Database License (http://opendatacommons.org/licenses/odbl/1.0/). The Open Database License (ODbL) is a license agreement intended to allow users to freely share, modify, and use this Dataset while maintaining this same freedom for others, provided that the original source and author(s) are credited.Hanh Thi Ngo, Quyen Hanh Do, Cuong The Pham, Vinh Quang Luu, L. Lee Grismer, Thomas Ziegler, Van Thi Hong Nguyen, Truong Quang Nguyen, Minh Duc Le

Supplementary material 4 Table S4 Data type Docx file. Explanation note Uncorrected (“p”) distance matrix showing percentage genetic divergence (COI) (highlighted in bold are the lowest and highest percentage) between species in the *Cyrtodactyluscondorensis* group. https://binary.pensoft.net/file/677234This dataset is made available under the Open Database License (http://opendatacommons.org/licenses/odbl/1.0/). The Open Database License (ODbL) is a license agreement intended to allow users to freely share, modify, and use this Dataset while maintaining this same freedom for others, provided that the original source and author(s) are credited.Hanh Thi Ngo, Quyen Hanh Do, Cuong The Pham, Vinh Quang Luu, L. Lee Grismer, Thomas Ziegler, Van Thi Hong Nguyen, Truong Quang Nguyen, Minh Duc Le

Supplementary material 5 Table S5 Data type Docx file. Explanation note Uncorrected (“p”) distance matrix showing percentage genetic divergence (COI) (highlighted in bold are the lowest and highest percentage) between species in the *Cyrtodactyluschauquangensis* group. https://binary.pensoft.net/file/677235This dataset is made available under the Open Database License (http://opendatacommons.org/licenses/odbl/1.0/). The Open Database License (ODbL) is a license agreement intended to allow users to freely share, modify, and use this Dataset while maintaining this same freedom for others, provided that the original source and author(s) are credited.Hanh Thi Ngo, Quyen Hanh Do, Cuong The Pham, Vinh Quang Luu, L. Lee Grismer, Thomas Ziegler, Van Thi Hong Nguyen, Truong Quang Nguyen, Minh Duc Le

Supplementary material 6 Table S6 Data type Docx file. Explanation note Uncorrected (“p”) distance matrix showing percentage genetic divergence (COI) (highlighted in bold are the lowest and highest percentage) between species in the *C.brevipalmatus and C.intermedius* group. https://binary.pensoft.net/file/677236This dataset is made available under the Open Database License (http://opendatacommons.org/licenses/odbl/1.0/). The Open Database License (ODbL) is a license agreement intended to allow users to freely share, modify, and use this Dataset while maintaining this same freedom for others, provided that the original source and author(s) are credited.Hanh Thi Ngo, Quyen Hanh Do, Cuong The Pham, Vinh Quang Luu, L. Lee Grismer, Thomas Ziegler, Van Thi Hong Nguyen, Truong Quang Nguyen, Minh Duc Le
