# Supplementary material for: ﻿How many more species are out there? Current taxonomy substantially underestimates the diversity of bent-toed geckos (Gekkonidae, Cyrtodactylus) in Laos and Vietnam
Source: Zookeys. 2022 Apr 26;1097:135–52. doi: 10.3897/zookeys.1097.78127 (PMC9848914; doi:10.3897/zookeys.1097.78127)
Supplement: Supplementary material 1 — Table S1 [file zookeys-1097-135_article-78127__-s001.docx]

**Supplementary Table 1**. Samples used in this study

| **Voucher/Field**  **number** | **Species** | **Locality** | **GenBank accession no.** | **References** |
| --- | --- | --- | --- | --- |
| PMNH 2301 | *Cyrtodactylus battalensis* | - | MF169903 | Brennan et al. (2017) |
| USNM 042 | *Hemidactylus frenatus* | - | MH274074 | Mulcahy et al. (unplublished) |
| KIZ13689 | *C. badenensis* | Vietnam: Ba Den Mountain, Thach Tan Com., Tay Ninh Prov. | KF929505 | Nguyen et al. (2014) |
| Tr0001 | *C. badenensis* | Vietnam: Ba Den Mountain, Thach Tan Com., Tay Ninh Prov. | ON145835 | This study^1^ |
| Tr0003 | *C. badenensis* | Vietnam: Ba Den Mountain, Thach Tan Com., Tay Ninh Prov. | ON145836 | This study^1^ |
| VNUF R.2016.4 | *C. bansocensis* | Laos: Khammouane Prov. | ON145865 | This study^2^ |
| VFU R.2015.21 | *C. bansocensis* | Laos: Ban Soc Village, Bualapha Dist. Khammouane Prov. | KU175573 | Luu et al. (2016) |
| NOUL.R-2015.21 | *C. bansocensis* | Laos: Ban Soc Village, Bualapha Dist. Khammouane Prov. | KU175574 | Luu et al. (2016) |
| TBU PAT250 | *C.* cf. *bichnganae* | Vietnam: Chieng Ngan Com., Chieng Sinh Dist., Son La City | KT004372 | Nguyen et al. (2015) |
| CS02.2015 | *C.* cf. *bichnganae* | Vietnam: Chieng Sinh Dist., Son La City | MF662968 | Pham et al. (2017) |
| CP2015.67 | *C.* cf. *bichnganae* | Vietnam: Chieng Pac Com., Thuan Chau Dist., Son La Prov. | MF662969 | Pham et al. (2017) |
| CX2015.14 | *C.* cf. *bichnganae* | Vietnam: Chieng Xom Com., Chieng Sinh Dist., Son La City | MF662970 | Pham et al. (2017) |
| PAT250 | *C.* cf. *bichnganae* | Vietnam: Son La City, Son La Prov. | ON145850 | This study^3^ |
| ITBCZ1537 | *C. bidoupimontis* | Vietnam: Bi Doup-Nui Ba NP, Lam Dong Prov. | KF169959 | Nguyen et al. (2013) |
| ZMMU NAP-00080 | *C. bidoupimontis* | Vietnam: Bi Doup-Nui Ba NP, Lam Dong Prov. | HQ967215 | Poyarkov et al. (unpublished) |
| CP17.01 | *C. bobrovi* | Vietnam: Cuc Phuong NP, Ninh Binh Prov. | ON145854 | This study^2^ |
| HB2015.73 | *C. bobrovi* | Vietnam: Tan Lac Dist., Hoa Binh Prov. | ON145853 | This study^1^ |
| IEBR A.2015.30 | *C. bobrovi* | Vietnam: Tan Lac Dist., Hoa Binh Prov. | KT004368 | Nguyen et al. (2015) |
| HB2015.83 | *C. bobrovi* | Vietnam: Tan Lac Dist., Hoa Binh Prov. | ON145855 | This study^1^ |
| VNMN A.2015.61 | *C. bobrovi* | Vietnam: Ngoc Son – Ngo Luong NR, Hoa Binh Prov. | KT004369 | Nguyen et al. (2015) |
| IEBR A.2011.3B | *C. bugiamapensis* | Vietnam: Bu Gia Map NP, Binh Phuoc Prov. | ON145810 | This study^1^ |
| VNMN994 | *C. bugiamapensis* | Vietnam: Bu Gia Map NP, Binh Phuoc Prov. | ON145811 | This study^2^ |
| ZMMU R-13093-2 | *C. bugiamapensis* | Vietnam: Bu Gia Map NP, Binh Phuoc Prov.s | HM888459 | Poyarkov et al. (unpublished) |
| NUOL.R-2015.22 | *C. calamei* | Laos: Thong Xam Village, Hian Nam No NPA, Khammouane Prov. | KX064043 | Luu et al. (2016) |
| VNUF R.2015.28 | *C. calamei* | Laos: Thong Xam Village, Hian Nam No NPA, Khammouane Prov. | KX064044 | Luu et al. (2016) |
| NT2016.2 | *C. caovansungi* | Vietnam: Ninh Hai Dist., Ninh Thuan Prov. | ON145812 | This study^1^ |
| NT2016.115 | *C. caovansungi* | Vietnam: Ninh Hai Dist., Ninh Thuan Prov. | ON145813 | This study^1^ |
| ITBCZ908 | *C. caovansungi* | Vietnam: Nui Chua NP., Ninh Thuan Prov. | KF219678 | Ziegler et al. (2013) |
| ITBCZ932 | *C. caovansungi* | Vietnam: Nui Chua NP., Ninh Thuan Prov. | KF219679 | Ziegler et al. (2013) |
| ZFMK88090 | *C. cattienensis* | Vietnam: Cat Tien NP, Dong Nai Prov. | ON145816 | This study^5^ |
| ZFMK88095 | *C. cattienensis* | Vietnam: Cat Tien NP, Dong Nai Prov. | ON145817 | This study^5^ |
| BPRes004 | *C. cattienensis* | Vietnam: Dong Phuoc Dist., Binh Phuoc Prov. | ON145818 | This study^4^ |
| BPRes007 | *C. cattienensis* | Vietnam: Dong Phuoc Dist., Binh Phuoc Prov. | ON145819 | This study^4^ |
| ZMMU NAP-00117.1 | *C. cattienensis* | Vietnam: Cat Tien NP, Nam Cat Tien, Dong Nai Prov. | HQ967220 | Poyarkov et al. (unpublished) |
| NA2016.1 | *C. chauquangensis* | Vietnam: Nghe An Prov. | MF957261 | Nguyen et al. (2017) |
| IEBR4581 | *C. chungi* | Vietnam: Binh Thuan Prov.^*^ | MT576019 | Ostrowski et al. (2021) |
| IEBR4582 | *C. chungi* | Vietnam: Binh Thuan Prov. | MT576020 | Ostrowski et al. (2021) |
| IEBR3269 | *C. condorensis* | Vietnam: Hon Dat Island, Kien Giang Prov. | ON145795 | This study^1^ |
| HMD KG2013.1 | *C. condorensis* | Vietnam: Hang Pagoda, Kien Giang Prov. | ON145796 | This study^4^ |
| Tr12 | *C. condorensis* | Vietnam: Hon Son Island, Ninh Hai Dist., Kien Giang Prov. | ON145800 | This study^6^ |
| IEBR R.4981 | *C. condorensis* | Vietnam: Hon Me Island, Kien Giang Prov. | ON145797 | This study^7^ |
| ITBCZ2605 | *C. condorensis* | Vietnam: Con Dao Island, Ba Ria – Vung Tau Prov. | ON145801 | This study^8^ |
| ITBCZ2553 | *C. condorensis* | Vietnam: Con Dao Island, Ba Ria – Vung Tau Prov. | ON145802 | This study^8^ |
| KIZ1022 | *C. condorensis* | Vietnam: Hon Chong Island, Kien Giang Prov. | KF929524 | Nguyen et al. (2014) |
| KIZ1023 | *C. condorensis* | Vietnam: Hon Chong Island, Kien Giang Prov. | KF929525 | Nguyen et al. (2014) |
| LSUHC 11397 | *C. condorensis* | Vietnam: Hon Thom Island, Kien Giang Prov. | ON145798 | This study^9^ |
| LSUHC 8596 | *C. condorensis* | Vietnam: Hon Son Island, Kien Giang Prov. | ON145799 | This study^9^ |
| KG2013.1 | *C. condorensis* | Vietnam: Kien Giang Prov. | ON145797 | This study^4^ |
| VNUF A.2014.69 | *C. cryptus* | Laos: Khammouane Prov. | KX064038 | Luu et al. (2016) |
| PNKB3 | *C. cryptus* | Vietnam: Phong Nha – Ke Bang NP, Quang Binh Prov. | KF169971 | Nguyen et al. (2013) |
| PNKB4 | *C. cryptus* | Vietnam: Phong Nha – Ke Bang NP, Quang Binh Prov. | KF169972 | Nguyen et al. (2013) |
| VNMN PMT 2142 | *C. cucdongensis* | Vietnam: Dak Nong Prov. | ON145814 | This study^6^ |
| IEBR A.2013.104 | *C. cucdongensis* | Vietnam: Cuc Dong Cape, Ninh Hoa District, Khanh Hoa Prov. | KJ403846 | Schneider et al. (2014) |
| ZFMK 95513 | *C. cucdongensis* | Vietnam: Cuc Dong Cape, Ninh Hoa District, Khanh Hoa Prov. | KJ403847 | Schneider et al. (2014) |
| UNS 0406 | *C. cucphuongensis* | Vietnam: Cuc Phuong NP, Ninh Binh Prov. | MF169912 | Brennan et al. (2017) |
| CP17.02 | *C. cucphuongensis* | Vietnam: Cuc Phuong NP, Ninh Binh Prov. | ON145856 | This study^2^ |
| NHQ.17.71 | *C. cucphuongensis* | Vietnam: Cuc Phuong NP, Ninh Binh Prov. | ON145857 | This study^2^ |
| VNMN04109 | *C. culaochamensis* | Vietnam: Cu Lao Cham Island, Quang Nam Prov. | ON145827 | This study^10^ |
| VNMN04118 | *C. culaochamensis* | Vietnam: Cu Lao Cham Island, Quang Nam Prov. | ON145828 | This study^10^ |
| ITBCZ2537 | *C. dati* | Vietnam: Bu Dop, Binh Phuong Prov. | KF929508 | Nguyen et al. (2014) |
| ITBCZ2539 | *C. dati* | Vietnam: Bu Dop, Binh Phuong Prov. | KF929510 | Nguyen et al. (2014) |
| ITBCZ2540 | *C. dati* | Vietnam: Bu Dop, Binh Phuong Prov. | KF929511 | Nguyen et al. (2014) |
| ZISP FN 256 | *C.* *darevskii* | Laos | HQ967221 | Orlov et al. (unpublished) |
| ZISP FN 187 | *C.* *darevskii* | Laos | HQ967223 | Orlov et al. (unpublished) |
| HNN16.11 | *C.* *darevskii* | Laos: Khammouane Prov. | ON145869 | This study^2^ |
| HNN16.38 | *C.* *darevskii* | Laos: Khammouane Prov. | ON145870 | This study^2^ |
| LSUHC 8597 | *C. eisenmanae* | Vietnam: Hon Son Island, Kien Giang Prov. | ON145808 | This study^9^ |
| LSUHC 8598 | *C. eisenmanae* | Vietnam: Hon Son Island, Kien Giang Prov. | ON145809 | This study^9^ |
| VNUF R.2017.4 | *C. gialaiensis* | Vietnam: Chu Se Dist., Gia Lai Prov. | MG460300 | Luu et al. (2017) |
| VNUF R.2017.1 | *C. gialaiensis* | Vietnam: Chu Se Dist., Gia Lai Prov.^*^ | MG460299 | Luu et al. (2017) |
| TZ68 | *C. grismeri* | Vietnam | ON145807 | This study^7^ |
| LSUHC 8639 | *C. grismeri* | Vietnam: Tuc Dup Hill, An Giang Prov. | ON145805 | This study^9^ |
| LSUHC 8638 | *C. grismeri* | Vietnam: Tuc Dup Hill, An Giang Prov. | ON145806 | This study^9^ |
| ITBCZ690 | *C. grismeri* | Vietnam: Tuc Dup Hill, An Giang Prov. | KF929515 | Nguyen et al. (2014) |
| IEBR A.2013.90 | *C. hinnamnoensis* | Laos: Ban Dou Village, Hin Nam No NPA, Khammouane Prov. | KX064046 | Luu et al. (2016) |
| IEBR A.2013.89 | *C. hinnamnoensis* | Laos: Khammouane Prov. | KJ817429 | Schneider et al. (2014) |
| KG2014.76 | *C. hontreensis* | Vietnam: Hon Me Island, Kien Giang Prov. | ON145839 | This study^1^ |
| KG2014.77 | *C. hontreensis* | Vietnam: Hon Me Island, Kien Giang Prov. | ON145840 | This study^1^ |
| IEBR A.2013.109 | *C. houaphanensis* | Laos: Houaphan Prov.^*^ | KJ817428 | Schneider et al. (2014) |
| IEBR A2013.3A | *C. huongsonensis* | Vietnam: Huong Son Mt., Hanoi | KX430034 | Le et al. (2016) |
| HB2016.32 | *C. huongsonensis* | Vietnam: Lac Thuy Dist., Hoa Binh Prov. | ON145844 | This study^1^ |
| HB2016.51 | *C. huongsonensis* | Vietnam: Lac Thuy Dist.Hoa Binh Prov. | ON145845 | This study^1^ |
| DN2017.1 | *C. huynhi* | Vietnam: Dong Nai Prov. | ON145831 | This study^1^ |
| DN2017.2 | *C. huynhi* | Vietnam: Dong Nai Prov. | ON145831 | This study^1^ |
| ITBCZ513 | *C. huynhi* | Vietnam: Chua Chan Mountain, Dong Nai Prov. | KF169948 | Nguyen et al. (2013) |
| TZ71 | *C. intermedius* | Vietnam | ON145837 | This study^7^ |
| ITBCZ609 | *C. intermedius* | Vietnam: Nui Cam, An Giang Prov. | KF169980 | Nguyen et al. (2013) |
| ITBCZ-10023 | *C. irregularis* | Vietnam: Lac Duong, Lam Dong Prov. | KP199951 | Poyarkov et al. (unpublished) |
| ITBCZ-10016 | *C. irregularis* | Vietnam: Lac Duong, Lam Dong Prov. | KP199952 | Poyarkov et al. (unpublished) |
| IEBR A.2013.55 | *C. jaegeri* | Laos: Thakhek, Khammouane Prov. | KT004364 | Nguyen et al. (2015) |
| VNUF R.2014.9 | *C. jaegeri* | Laos: Thakhek, Khammouane Prov. | KT004366 | Nguyen et al. (2015) |
| IEBR A.2013.55-1 | *C. jaegeri* | Laos: Thakhek, Khammouane Prov. | ON175939 | This study^1^ |
| VNUF R.2015.7 | *C.* *jarujini* | Laos: Borikhamxay Prov. | KX077907 | Luu et al. (2016) |
| PKK16.01 | *C. jarujini* | Laos: Borikhamxay Prov. | ON145876 | This study^2^ |
| IEBR A.2013.3 | *C. kingsadai* | Vietnam: Khanh Hoa Prov. | KF188432 | Ziegler et al. (2013) |
| VNMN PMT 2134 | *C. kingsadai* | Vietnam: Dak Nong Prov. | ON145833 | This study^6^ |
| IEBR A.2013.3-2 | *C. kingsadai* | Vietnam: Dai Lanh Cape, Phu Yen Prov. | ON145834 | This study^1^ |
| ZISP FN 191 | *C. khammouanensis* | Laos | HḾ́́888467 | Orlov et al. (unpublished) |
| ZISP FN 257 | *C. khammouanensis* | Laos | HM888469 | Orlov et al. (unpublished) |
| LSUHC 11376 | *C. leegrismeri* | Vietnam: Hon Khoai Island, Ca Mau Prov. | ON145803 | This study^9^ |
| LSUHC 11410 | *C. leegrismeri* | Vietnam: Hon Tho Chau Island, Kien Giang Prov. | ON145804 | This study^9^ |
| UNS0527 | *C. lomyenensis* | Laos: Khammouane Prov. | ON145866 | This study^11^ |
| UNS 0471 | *C. martini* | Vietnam: Lai Chau Prov.^*^ | MF169929 | Brennan et al. (2017) |
| VNUF R.2018.32 | *C. muangfuangensis* | Laos: Vientiane Prov. | MN395826 | Janssen et al. (2019) |
| NUOL R.2018.33 | *C. muangfuangensis* | Laos: Vientiane Prov. | MN395827 | Janssen et al. (2019) |
| ZISP FN 2 | *C. multiporus* | Laos | HM888471 | Orlov et al. (unpublished) |
| ZISP FN 3 | *C. multiporus* | Laos | HM888472 | Orlov et al. (unpublished) |
| VNUF R.2014.52 | *C. multiporus* | Laos: Khammouane Prov. | ON145874 | This study^2^ |
| VNUF R.2014.23 | *C. multiporus* | Laos: Khammouane Prov. | ON145875 | This study^2^ |
| IEBR 4829 | *C. ngati* | Vietnam: Pa Thom, Dien Bien Prov. | MW655789 | Le et al. (2021) |
| PT2017.215 | *C. ngati* | Vietnam: Dien Bien Prov. | ON145841 | This study^3^ |
| VNUF R.2014.50 | *C.* cf. *ngati* | Laos: Khammouane Prov. | KX077901 | Luu et al. (2016) |
| LPB62 | *C. ngoiensis* | Laos: Luang Prabang Prov. | ON145860 | This study^2^ |
| LPB63 | *C. ngoiensis* | Laos: Luang Nam Tha Prov. | ON145861 | This study^2^ |
| LPB62-1 | *C. ngoiensis* | Laos: Luang Prabang Prov. | ON175940 | This study^2^ |
| AT2012.1 | *C. ngoiensis* | Laos: Luang Prabang Prov. | KJ817432 | Schneider et al. (2019) |
| VNMN2184 | *C. nigriocularis* | Vietnam: Ba Den Mountain, Thach Tan Com., Tay Ninh Prov. | ON145793 | This study^10^ |
| VNMN2189 | *C. nigriocularis* | Vietnam: Ba Den Mountain, Thach Tan Com., Tay Ninh Prov. | ON145794 | This study^10^ |
| VNMN2187 | *C. nigriocularis* | Vietnam: Ba Den Mountain, Thach Tan Com., Tay Ninh Prov. | KF929523 | Nguyen et al. (2014) |
| IEBR 3811 | *C. orlovi* | Vietnam: Ninh Thuan Prov. | MZ440851 | Do et al. (2021) |
| IEBR A.2015.26 | *C. otai* | Vietnam: Hang Kia-Pa Co, Mai Chau Dist., Hoa Binh Prov. | KT004370 | Nguyen et al . (2015) |
| IEBR A.2015.27 | *C. otai* | Vietnam: Hang Kia-Pa Co, Mai Chau Dist., Hoa Binh Prov. | KT004371 | Nguyen et al. (2015) |
| TBU 2017.2 | *C. otai* | Vietnam: Chieng Yen Com., Van Ho Dist., Son La Prov. | MF957262 | Nguyen et al. (2017) |
| IEBR A.2017.3 | *C. otai* | Vietnam: Chieng Yen Com., Van Ho Dist., Son La Prov. | MF957263 | Nguyen et al. (2017) |
| ZFMK 91827 | *C. pageli* | Laos: Vientiane Prov. | KJ817431 | Schneider et al. (2014) |
| NQT2010.36 | *C.* cf. *pageli* | Laos: Vientiane Prov. | ON145867 | This study^1^ |
| ZFMK91827 | *C. pageli* | Laos: Vientiane Prov. | ON145868 | This study^1^ |
| CBC 3003 | *C. phnomchiensis* | Cambodia: Prey Lang Wildlife Sanctuary, Phnom Chi, Sadan Dist. Kampong Thom Prov. | MT066405 | Neang et al. (2020) |
| CBC 3004 | *C. phnomchiensis* | Cambodia: Prey Lang Wildlife Sanctuary, Phnom Chi, Sadan Dist. Kampong Thom Prov. | MT066406 | Neang et al. (2020) |
| PNKN201130 | *C. phongnhakebangensis* | Vietnam: Phong Nha-Ke Bang NP, Quang Binh Prov. | KF929526 | Nguyen et al. (2014) |
| PNKN201132 | *C. phongnhakebangensis* | Vietnam: Phong Nha-Ke Bang NP, Quang Binh Prov. | KF929527 | Nguyen et al. (2014) |
| ZFMK 103153 | *C. phumyensis* | Vietnam: Phu My Dist., Binh Dinh Prov. | MT210158 | Ostrowski et al. (2020) |
| IEBR 4577 | *C. phumyensis* | Vietnam: Phu My Dist., Binh Dinh Prov.^*^ | MT210159 | Ostrowski et al. (2020) |
| PQ.2015.15 | *C. phuquocensis* | Vietnam: Phu Quoc Island, Kien Giang Prov. | ON145838 | This study^1^ |
| KH–Res041 | *C. phuocbinhensis* | Vietnam: O Kha Valley, Khanh Son Dist., Khanh Hoa Prov. | ON145822 | This study^4^ |
| KH–Res042 | *C. phuocbinhensis* | Vietnam: O Kha Valley, Khanh Son Dist., Khanh Hoa Prov. | ON145823 | This study^4^ |
| ITBCZ1518 | *C. phuocbinhensis* | Vietnam: Phuoc Binh NP., Ninh Thuan Prov. | KF169953 | Nguyen et al. (2013) |
| ITBCZ1529 | *C. phuocbinhensis* | Vietnam: Phuoc Binh NP., Ninh Thuan Prov.^*^ | KF169954 | Nguyen et al. (2013) |
| ITBCZ3001 | *C.* cf. *pseudoquadrivirgatus* | Vietnam: A Luoi Dist., Thua Thien Hue Prov. | KF169963 | Nguyen et al. (2013) |
| ITBCZ3002 | *C.* cf. *pseudoquadrivirgatus* | Vietnam: A Luoi Dist., Thua Thien Hue Prov. | KF169964 | Nguyen et al. (2013) |
| AL.2017.125 | *C.* cf. *pseudoquadrivirgatus* | Vietnam: A Luoi Dist., Thua Thien Hue Prov. | ON145824 | This study^1^ |
| AL.2017.126 | *C.* cf. *pseudoquadrivirgatus* | Vietnam: A Luoi Dist., Thua Thien Hue Prov. | ON145825 | This study^1^ |
| ZMMU R-13095-2 | *C.* cf. *pseudoquadrivirgatus* | Vietnam | KP199949 | Poyarkov et al. (unpublished) |
| ZMMU R130952 | *C.* cf. *pseudoquadrivirgatus* | - | MG791888 | Pauwels et al. (2018) |
| ITBCZ2532 | *C.* cf. *pseudoquadrivirgatus* | Vietnam: Ba Na Resort, Da Nang City | KF169962 | Nguyen et al. (2013) |
| TIBCZ 2530 | *C.* cf. *pseudoquadrivirgatus* | Vietnam: Ba Na Resort, Da Nang City | KY862137 | Nguyen et al. (2017) |
| ND01.15 | *C. puhuensis* | Vietnam: Thanh Hoa Prov. | ON145851 | This study^2^ |
| ND02.15 | *C. puhuensis* | Vietnam: Thanh Hoa Prov. | ON145852 | This study^2^ |
| KIZ11665 | *C. puhuensis* | Vietnam: Pu Hu NR., Thanh Hoa Prov. | KF929529 | Nguyen et al. (2014) |
| PNKB20113 | *C. roesleri* | Vietnam: Phong Nha-Ke Bang NP., Quang Binh Prov. | KF929531 | Nguyen et al. (2014) |
| PNKB201134 | *C. roesleri* | Vietnam: Phong Nha-Ke Bang NP., Quang Binh Prov. | KF929532 | Nguyen et al. (2014) |
| ZFMK 89377 | *C. roesleri* | Vietnam: Phong Nha-Ke Bang NP., Quang Binh Prov. | ON145862 | This study^7^ |
| ZFMK 89378 | *C. roesleri* | Vietnam: Phong Nha-Ke Bang NP., Quang Binh Prov. | ON145863 | This study^7^ |
| VFU R.2015.14 | *C. rufford* | Laos: Khammouane Prov.^*^ | KU175572 | Luu et al. (2016) |
| ITBCZ 995 | *C. sangi* | Vietnam: Nui Chua NP., Thuan Bac Dist., Ninh Thuan Prov. | KY862153 | Nguyen et al. (2017) |
| ZMMU R-11503-2 | *C. sangi* | Vietnam: Nui Chua NP., Thuan Bac Dist., Ninh Thuan Prov. | KC016080 | Nazarov et al. (2012) |
| NT.2016.1 | *C. sangi* | Vietnam: Ninh Hai Dist., Ninh Thuan Prov. | ON145820 | This study^1^ |
| NT.2016.114 | *C. sangi* | Vietnam: Ninh Hai Dist., Ninh Thuan Prov. | ON145821 | This study^1^ |
| IEBR A.2013.112 | *C. sommerladi* | Laos: Khammouane Prov. | KJ817437 | Schneider et al. (2014) |
| VNUF R.2014.87 | *C. sommerladi* | Laos: Khammouane Prov. | ON145864 | This study^2^ |
| HNUE VL.2015.78 | *C. soni* | Vietnam: Van Long Wetland NR., Ninh Binh Prov.^*^ | KX430032 | Le et al. (2016) |
| IEBR R.2016.4 | *C. soni* | Vietnam: Van Long Wetland NR., Ninh Binh Prov. | KX430033 | Le et al. (2016) |
| CP2016.23 | *C. soni* | Vietnam: Cuc Phuong NP., Ninh Binh Prov. | ON145846 | This study^2^ |
| CP2016.24 | *C. soni* | Vietnam: Cuc Phuong NP., Ninh Binh Prov. | ON145847 | This study^2^ |
| HN17.01 | *C. soni* | Vietnam: Ha Nam Prov. | ON145848 | This study^2^ |
| HN17.06 | *C. soni* | Vietnam: Ha Nam Prov. | ON145849 | This study^2^ |
| IEBR A.2017.1 | *C. sonlaensis* | Vietnam: Muong Bang Com., Phu Yen Dist., Son La Prov.^*^ | MF957264 | Nguyen et al. (2017) |
| IEBR A.2017.2 | *C. sonlaensis* | Vietnam: Muong Bang Com., Phu Yen Dist., Son La Prov. | MF957265 | Nguyen et al. (2017) |
| NUOL R-2015.5 | *C. soudthichaki* | Laos: Khun Don, Khammouane Prov. | KX077904 | Luu et al. (2016) |
| VFU R.2015.18 | *C. soudthichaki* | Laos: Khun Don, Khammouane Prov. | KX077905 | Luu et al. (2016) |
| ZMMU R-13980-3 | *C. spelaeus* | Laos: Kasi Dist., Vientiane Prov. | KP199947 | Poyarkov et al. (unpublished) |
| ZMMU R-13980-1 | *C. spelaeus* | Laos: Kasi Dist, Vientiane Prov. | KP199948 | Poyarkov et al. (unpublished) |
| ITBCZ2528 | *C. takouensis* | Vietnam: Ta Kou NR., Binh Thuan Prov. | KF929534 | Nguyen et al. (2014) |
| ITBCZ2527 | *C. takouensis* | Vietnam: Ta Kou NR., Binh Thuan Prov. | KF929533 | Nguyen et al. (2014) |
| IEBR 4379 | *C. taybacensis* | Vietnam: Son La Prov. | MH997990 | Pham et al. (2019) |
| TBU 09 | *C. taybacensis* | Vietnam: Son La Prov. | MH997989 | Pham et al. (2019) |
| ROM32119 | *C. taynguyenensis* | Vietnam: Krongpa Village, Gia Lai Prov. | KF169978 | Nguyen et al. (2013) |
| ROM32120 | *C. taynguyenensis* | Vietnam: Krongpa Village, Gia Lai Prov.^*^ | KF169979 | Nguyen et al. (2013) |
| KM2012.77 | *C. teyniei* | Laos: Khammouane Prov. | KJ817430 | Schneider et al. (2014) |
| Lak Sao16.01 | *C. teyniei* | Laos: Borikhamxay Prov. | ON145873 | This study^2^ |
| KM2012.77 | *C. teyniei* | Laos: Borikhamxay Prov. | ON145872 | This study^2^ |
| ZMMU R-14919-1 | *C. thathomensis* | Laos: Ban Thathom, Xiangkhoang Prov.^*^ | MG791873 | Nazarov et al. (2018) |
| ZMMU R-14919-3 | *C. thathomensis* | Laos: Ban Thathom, Xiangkhoang Prov. | MG791875 | Nazarov et al. (2018) |
| IEBR A2013.23 | *C. thuongae* (*dati*) | Vietnam: Ba Den Mountain, Thach Tan Com., Tay Ninh Prov. | ON145829 | This study^1^ |
| IEBR A2013.25 | *C. thuongae* (*dati*) | Vietnam: Ba Den Mountain, Thach Tan Com., Tay Ninh Prov. | ON145830 | This study^1^ |
| NUOL R-2013.5 | *C. vilaphongi* | Laos: Ban Xieng Muak, Luang Prabang Dist., Luang Prabang Prov. | KJ817434 | Schneider et al. (2014) |
| IEBR A.2013.103 | *C. vilaphongi* | Laos: Ban Xieng Muak, Luang Prabang Dist., Luang Prabang Prov. | KJ817435 | Schneider et al. (2014) |
| LPB 46 | *C. vilaphongi* | Laos: Luang Prabang Prov. | ON145858 | This study^2^ |
| LPB 68 | *C. vilaphongi* | Laos: Luang Prabang Prov. | ON145859 | This study^2^ |
| ZMMU R-13981-1 | *C. wayakonei* | Laos: Luang Namtha Prov. | KP199950 | Poyarkov et al. (unpublished) |
| ZFMK91016 | *C. wayakonei* | Laos: Luang Namtha Prov. | KJ817438 | Schneider et al. (2014) |
| ITBCZ 3540 | *C. yangbayensis* | Vietnam: Khanh Hoa | MG652461 | Nguyen et al. (2017) |
| UNS 0476 | *C. yangbayensis* | Vietnam: Yang Bay Waterfall, Khanh Hoa Prov.^*^ | MF169952 | Brennan et al. (2017) |
| ITBCZ 3541 | *C. yangbayensis* | Vietnam: Yang Bay Waterfall, Khanh Hoa Prov. | ON145815 | This study^8^ |
| ZMMU R-13116-3 | *C.* cf. *ziegleri* | Vietnam: Chu Yang Sin NP., Dak Lak Prov. | HQ967210 | Orlov et al. (unpublished) |
| UNS 5007 | *C.* cf. *ziegleri* | Vietnam: Chu Yang Sin NP., Dak Lak Prov. | KF169945 | Nguyen et al. (2013) |
| VNMN2014 | *C.* cf. *ziegleri* | Vietnam: Na Nung, Dak Nong Prov. | KF169975 | Nguyen et al. (2013) |
| VNMN 2015 | *C.* cf. *ziegleri* | Vietnam: Na Nung, Dak Nong Prov. | KF169976 | Nguyen et al. (2013) |
| KM2012.52 | *C.* sp. 1 | Laos: Khammouane Prov. | KP199942 | Poyarkov et al. (unpublished) |
| KM2012.54-1 | *C.* sp. 1 | Laos: Khammouane Prov. | KJ817436 | Schneider et al. (2014) |
| KM2012.54-2 | *C.* sp. 1 | Laos: Khammouane Prov. | ON145871 | This study^2^ |
| ITBCZ 1502 | *C.* sp. 2 | Vietnam: Mdrak, Dak Lak Prov. | KY862148 | Nguyen et al. (2017) |
| ITBCZ1507 | *C.* sp. 2 | Vietnam: Mdrak, Dak Lak Prov. | KY862149 | Nguyen et al. (2017) |
| ITBCZ1512 | *C.* sp. 2 | Vietnam: Mdrak, Dak Lak Prov. | KY862151 | Nguyen et al. (2017) |
| ITBCZ491 | *C.* sp. 3 | Vietnam: Nui Dinh, Ba Ria-Vung Tau Prov. | KY862254 | Nguyen et al. (2017) |
| UNS 0473 | *C.* sp. 3 | Vietnam: Son La Urban, Son La Prov. | MF169904 | Brennan et al. (2017) |
| YPX18356 | *C.* sp. 4 | Vietnam: Bac Huong Hoa, Quang Tri Prov. | KY862129 | Nguyen et al. (2017) |
| KIZ010100 | *C.* cf. sp. 4 | Vietnam: Phong Dien, Thua Thien Hue Prov. | KY862131 | Nguyen et al. (2017) |
| KIZ010691 | *C.* cf. sp. 4 | Vietnam: Phong Dien, Thua Thien Hue Prov. | KY862132 | Nguyen et al. (2017) |
| QB.2015.325 | *C.* sp. 4 | Vietnam: Quang Binh Prov. | ON145826 | This study^1^ |
| KIZ013699 | *C.* sp. 5 | Vietnam: Suoi Luong, Da Nang City | KY862133 | Nguyen et al. (2017) |
| KIZ013700 | *C.* sp. 5 | Vietnam: Suoi Luong, Da Nang City | KY862134 | Nguyen et al. (2017) |
| VNMN3372 | *C.* sp. 6 | Vietnam: Ba To, Quang Ngai Prov. | KY862141 | Nguyen et al. (2017) |
| ROM30541 | *C.* sp. 6 | Vietnam: Tram Lap, Gia Lai Prov. | KY862143 | Nguyen et al. (2017) |
| CS02.2015 | *C.* sp. 6 | Vietnam: Chieng Sinh Dist., Son La City | MF662968 | Pham et al. (2017) |
| KIZ201103 | *C.* sp. 7 | Vietnam | KF929537 | Nguyen et al. (2014) |

**Notes.** Asterisks in the thirst column indicate type localities. ^1^ Specimens were collected by Nguyen et al. (IEBR), ^2^ Specimens were collected by Luu et al. (VNUF), ^3^ Specimens were collected by Pham et al. (University of Sciences, Hanoi, Vietnam), ^4^ Specimens were collected by Nguyen HN, ^5^ Specimens were collected by Geissler P, ^6^ Specimens were collected by Phung TM, ^7^ Specimens were collected by Ziegler et al., ^8^ Specimens were collected by Nguyen et al. (Institute of Tropical Biology, Ho Chi Minh City, Vietnam), ^9^ Specimens were collected by Grismer et al., ^1 0^Specimens were collected by Nguyen et al. (VNMN), ^11^ Specimens were collected by Ngo TV (UNS).

**Field numbers are assigned to following voucher numbers:**

KG2014.8 = IEBR R.4981 HB2016.51 = IEBR R.4983 HNN16.38 = VNUF R.2016.38

NT2016.2 = IEBR R.4979 CP2016.23 = CPNP R.2016.23 Lak Sao16.01 = VNUF R.2016.01

NT2016.115 = IEBR R.4980 CP2016.24 = CPNP R.2016.24 HNN52.14 = VNUF R.2014.52

NT.2016.1 = IEBR R.4988 HN17.01 = VNUF. RHN.2017.01 HNN23.14 = VNUF R.2014.23

NT.2016.114 = IEBR R.4989 HN17.06 = VNUF. RHN.2017.06 PKK16.01 = VNUF R.2016.01

AL.2017.125 = IEBR R.4986 ND01.15 = VNUF R.2015.01 PT2017.215 = HNUE-R00111

AL.2017.126 = IEBR R.4987 ND02.15 = VNUF R.2015.02 HNN87.14 = VNUF R.2014.87

DN2017.1 = IEBR R.4984 HB2015.73 = IEBR R.4977

DN2017.2 = IEBR R.4985 HB2015.83 = IEBR R.4978

Tr0007 = IEBR A.2013.3-2 CP17.01 = CPNP R.2017.122

Tr0001 = IEBR R.4975 HNN16.4 = VNUF R.2016.4

Tr0003 = IEBR R.4976 NQT2010.36 = IEBR A.2010.1

HB2016.32 = IEBR R.4982 HNN16.11 = VNUF R.2016.11
