# Supplementary material for: ﻿How many more species are out there? Current taxonomy substantially underestimates the diversity of bent-toed geckos (Gekkonidae, Cyrtodactylus) in Laos and Vietnam
Source: Zookeys. 2022 Apr 26;1097:135–52. doi: 10.3897/zookeys.1097.78127 (PMC9848914; doi:10.3897/zookeys.1097.78127)
Supplement: Supplementary material 2 — Table S2 [file zookeys-1097-135_article-78127__-s002.docx]

**Supplementary Table 2.** Uncorrected (“p”) distance matrix showing percentage genetic divergence (COI) (highlighted in bold are the lowest and highest percentage) between species in the *Cyrtodactylus angularis* group.

|  | 1 | 2 | 3 | 4 | 5 | 6 | 7 | 8 | 9 | 10 | 11 | 12 | 13 | 14 | 15 | 16 | 17 | 18 | 19 | 20 | 21 |
| --- | --- | --- | --- | --- | --- | --- | --- | --- | --- | --- | --- | --- | --- | --- | --- | --- | --- | --- | --- | --- | --- |
| 1*. C. bansocensis* VNUF R.2016.4 | - |  |  |  |  |  |  |  |  |  |  |  |  |  |  |  |  |  |  |  |  |
| 2*. C. calamei* NUOL.R-2015.22 | 16.13 | - |  |  |  |  |  |  |  |  |  |  |  |  |  |  |  |  |  |  |  |
| 3. *C. darevskii* VNUF R.2016.38 | 17.00 | 4.39 | - |  |  |  |  |  |  |  |  |  |  |  |  |  |  |  |  |  |  |
| 4. *C. hinnamnoensis* VNUF A.2015.3 | 17.20 | 5.33 | 3.76 | - |  |  |  |  |  |  |  |  |  |  |  |  |  |  |  |  |  |
| 5. *C. jaegeri* IEBR A.2013.55 | 15.07 | 16.44 | 17.15 | 17.96 | - |  |  |  |  |  |  |  |  |  |  |  |  |  |  |  |  |
| 6. *C. jarujini* VNUF R.2015.7 | 17.81 | 16.59 | 16.32 | 16.29 | 17.81 | - |  |  |  |  |  |  |  |  |  |  |  |  |  |  |  |
| 7. *C. khammouanensis* ZISP FN191 | 12.18 | 13.85 | 15.20 | 16.59 | 14.76 | 15.98 | - |  |  |  |  |  |  |  |  |  |  |  |  |  |  |
| 8. *C. lomyenensis* UNS0527 | 11.87 | 15.07 | 17.28 | 18.57 | 15.22 | 17.50 | 11.87 | - |  |  |  |  |  |  |  |  |  |  |  |  |  |
| 9. *C. muangfuangensis* VNUF R.20.18.32 | 18.72 | 18.27 | 19.40 | 19.18 | 19.33 | 21.16 | 21.16 | 18.27 | - |  |  |  |  |  |  |  |  |  |  |  |  |
| 10. *C. multiporus* ZISP FN 3 | 16.29 | 15.37 | 14.69 | 15.53 | 16.29 | 9.59 | 16.29 | 16.29 | 19.33 | - |  |  |  |  |  |  |  |  |  |  |  |
| 11. *C*. *multiporus* VNUF R.2014.23 | 16.29 | 15.53 | 14.36 | 15.22 | 16.13 | 10.35 | 16.29 | 16.74 | 18.87 | 1.37 | - |  |  |  |  |  |  |  |  |  |  |
| 12. *C. nigriocularis* VNMN2187 | 20.55 | 22.03 | 22.92 | **24.04** | 21.25 | 22.51 | 22.00 | 21.24 | 20.93 | 21.26 | 21.26 | - |  |  |  |  |  |  |  |  |  |
| 13. *C. pageli* ZFMK91827 | 19.33 | 17.81 | 18.64 | 19.03 | 19.18 | 17.50 | 18.42 | 18.11 | 19.94 | 17.20 | 16.90 | 22.22 | - |  |  |  |  |  |  |  |  |
| 14*. C. phongnhakebangensis* PNKN201132 | 15.77 | 7.86 | 8.56 | 9.34 | 16.70 | 17.04 | 15.65 | 17.22 | 17.97 | 15.27 | 15.11 | 21.82 | 17.66 | - |  |  |  |  |  |  |  |
| 15. *C. roesleri* ZFMK89377 | 15.37 | 15.22 | 16.65 | 17.20 | 14.76 | 17.96 | 16.29 | 15.83 | 18.42 | 17.20 | 16.90 | 20.13 | 17.50 | 15.59 | - |  |  |  |  |  |  |
| 16. *C. rufford* VFUR2015.14. | 11.42 | 15.37 | 17.59 | 18.87 | 14.61 | 17.66 | 12.02 | **2.44** | 18.11 | 16.59 | 17.20 | 20.68 | 18.72 | 17.57 | 16.13 | - |  |  |  |  |  |
| 17. *C. sommerladi* IEBR A.2013.112 | 15.68 | 14.61 | 15.68 | 16.29 | 15.83 | 17.81 | 16.59 | 15.98 | 19.33 | 16.90 | 16.59 | 20.70 | 16.44 | 16.35 | 6.09 | 17.20 | - |  |  |  |  |
| 18. *C.* sp. 1 KM2012.54 | 16.29 | 14.61 | 14.51 | 14.92 | 16.59 | 15.37 | 16.29 | 17.20 | 20.40 | 14.61 | 15.22 | 20.54 | 17.35 | 14.64 | 17.96 | 17.96 | 17.96 | - |  |  |  |
| 19. *C. soudthichaki* NUOL R-2015.5 | 12.63 | 17.05 | 17.80 | 18.87 | 14.16 | 18.27 | 13.39 | 14.00 | 20.40 | 17.66 | 17.35 | 21.24 | 18.27 | 17.81 | 15.22 | 14.46 | 15.53 | 18.57 | - |  |  |
| 20. *C. thathomensis* ZMMU R-14919-3 | 16.90 | 16.90 | 16.33 | 16.74 | 17.66 | 4.57 | 17.50 | 17.66 | 19.64 | 9.74 | 10.50 | 21.77 | 18.11 | 15.93 | 17.81 | 18.72 | 17.05 | 15.83 | 18.27 | - |  |
| 21. *C. teyniei* KM2012.77 | 18.15 | 14.15 | 14.83 | 15.52 | 17.63 | 9.26 | 16.67 | 17.07 | 20.55 | 6.59 | 7.42 | 22.19 | 17.44 | 15.30 | 18.28 | 17.55 | 17.94 | 14.59 | 16.76 | 9.76 | - |

Notes: The genetic divergences between samples of *C. bansocensis* are 0.15 – 0.61%; *C. calamei* are 0.15%; *C. darevskii* are 0.00 – 2.28%; *C. hinnamnoensis* are 2.28%; *C. jaegeri* are 0.00%; *C. jarujini* are 0.15%; *C. khammouanensis* are 0.15%; *C. muangfuangensis* are 0.30%; *C. multiporus* are 0.00 – 1.52%; *C. nigriocularis* are 0.00 – 1.84% ; *C. pageli* and *C.* cf. *pageli* are 0.00 – 6.2%; *C. phongnhakebangensis* are 0.00%; *C. roesleri* are 0.18 – 2.13%; *C. sommerladi* are 0.31%; *C. soudthichaki* are 0.15%; *C. thathomensis* are 0.00%; *C. teynei* are 0.00%.
