# Supplementary material for: ﻿How many more species are out there? Current taxonomy substantially underestimates the diversity of bent-toed geckos (Gekkonidae, Cyrtodactylus) in Laos and Vietnam
Source: Zookeys. 2022 Apr 26;1097:135–52. doi: 10.3897/zookeys.1097.78127 (PMC9848914; doi:10.3897/zookeys.1097.78127)
Supplement: Supplementary material 3 — Table S3 [file zookeys-1097-135_article-78127__-s003.docx]

**Supplementary Table 3.** Uncorrected (“p”) distance matrix showing percentage genetic divergence (COI) (highlighted in bold are the lowest and highest percentage) between species in the *Cyrtodactylus irregularis* group.

|  | 1 | 2 | 3 | 4 | 5 | 6 | 7 | 8 | 9 | 10 | 11 | 12 | 13 | 14 | 15 | 16 | 17 |
| --- | --- | --- | --- | --- | --- | --- | --- | --- | --- | --- | --- | --- | --- | --- | --- | --- | --- |
| 1. *C. badenensis* KIZ13689 | - |  |  |  |  |  |  |  |  |  |  |  |  |  |  |  |  |
| 2. *C. bidoupimontis* ZMMU NAP-00080 | 18.74 | - |  |  |  |  |  |  |  |  |  |  |  |  |  |  |  |
| 3. *C. bugiamapensis* IEBR A.2011.3B | 18.55 | 13.24 | - |  |  |  |  |  |  |  |  |  |  |  |  |  |  |
| 4. *C. caovansungi* IEBR R.4979 | 20.53 | 15.07 | 14.00 | - |  |  |  |  |  |  |  |  |  |  |  |  |  |
| 5. *C. cattienensis* ZFMK88090 | 19.79 | 15.83 | 15.83 | 16.74 | - |  |  |  |  |  |  |  |  |  |  |  |  |
| 6. *C chungi* IEBR4581 | 21.03 | 16.44 | 16.13 | 17.05 | 9.13 | - |  |  |  |  |  |  |  |  |  |  |  |
| 7. *C. cucdongensis* VNMN PMT2142 | 19.60 | 13.70 | 14.16 | 15.68 | 17.20 | 17.05 | - |  |  |  |  |  |  |  |  |  |  |
| 8. *C. culaochamensis* VNMN04109 | 16.29 | 13.70 | 13.70 | 13.85 | 13.85 | 15.22 | 14.46 | - |  |  |  |  |  |  |  |  |  |
| 9. *C. cryptus* VNUF A.2014.69 | 19.36 | 14.31 | 15.53 | 15.07 | 17.35 | 17.35 | 14.92 | 13.55 | - |  |  |  |  |  |  |  |  |
| 10. *C. dati* ITBCZ2537 | 17.82 | 14.71 | 15.05 | 17.07 | 18.33 | 18.31 | 17.04 | 15.20 | 16.78 | - |  |  |  |  |  |  |  |
| 11. *C. gialaiensis* VNUF R.2017.1 | 18.74 | 15.83 | 14.76 | 17.20 | 16.29 | 16.59 | 16.44 | 15.68 | 16.90 | 16.89 | - |  |  |  |  |  |  |
| 12. *C. huynhi* ITBCZ513 | 18.00 | 14.18 | 15.24 | 16.71 | 18.16 | 17.41 | 16.68 | 15.20 | 16.77 | 4.18 | 17.08 | - |  |  |  |  |  |
| 13. *C. irregularis* ITBCZ-10023 | 18.19 | 8.86 | 15.13 | 16.96 | 18.50 | 16.06 | 14.05 | 14.82 | 14.97 | 14.71 | 15.59 | 14.89 | - |  |  |  |  |
| 14. *C. kingsadai* IEBR A.2013.3 | 17.41 | 14.92 | 14.61 | 15.22 | 17.35 | 16.44 | 15.68 | 14.00 | 12.79 | 15.03 | 17.50 | 15.04 | 15.59 | - |  |  |  |
| 15. *C. orlovi* IEBR 3811 | 20.40 | 15.68 | 13.24 | 15.83 | 11.72 | 11.57 | 17.20 | 14.61 | 16.13 | 17.82 | 15.07 | 17.46 | 17.12 | 16.59 | - |  |  |
| 16. *C. phnomchiensis* CBC 3003 | 18.56 | 14.46 | 7.15 | 13.70 | 17.05 | 16.59 | 15.53 | 14.92 | 15.68 | 14.38 | 14.76 | 13.11 | 14.98 | 15.37 | 15.22 | - |  |
| 17*. C. phumyensis* ZFMK 103153 | 19.16 | 14.46 | 14.76 | 14.76 | 16.59 | 15.68 | 8.98 | 14.31 | 14.46 | 15.70 | 16.90 | 15.17 | 13.75 | 14.92 | 15.68 | 15.53 | - |
| 18. *C. phuocbinhensis* KH-Res041 | 18.17 | 13.39 | 13.55 | 14.46 | 14.61 | 14.31 | 14.00 | 12.02 | 13.70 | 15.45 | 14.31 | 15.99 | 13.44 | 12.94 | 14.31 | 14.00 | 13.55 |
| 19. *C. pseudoquadrivirgatus* ITBCZ3001 | 16.36 | 13.96 | 13.23 | 15.07 | 14.87 | 14.35 | 14.48 | 6.45 | 13.46 | 14.36 | 15.61 | 14.36 | 13.61 | 13.91 | 15.64 | 14.55 | 12.26 |
| 20. *C. pseudoquadrivirgatus* ZMMU R130952 | 17.51 | 13.29 | 15.17 | 16.13 | 16.85 | 16.34 | 16.03 | 9.60 | 15.57 | 15.74 | 15.18 | 14.90 | 13.62 | 15.77 | 16.65 | 14.62 | 13.43 |
| 21. *C.* cf. *pseudoquadrivirgatus* ITBCZ2532 | 18.73 | 15.74 | 16.26 | 15.19 | 18.48 | 17.20 | 16.40 | 14.72 | 9.01 | 16.36 | 17.12 | 15.64 | 15.36 | 14.40 | 18.84 | 15.39 | 15.45 |
| 22. *C. sangi* IEBR R.4988 | 19.40 | 15.22 | 14.61 | 16.13 | 12.48 | 13.09 | 15.22 | 14.16 | 14.61 | 17.96 | 15.22 | 17.42 | 16.35 | 15.22 | 12.94 | 15.07 | 15.98 |
| 23*. Cyrtodactylus* cf. sp. 2 ITBCZ1502 | 17.46 | 13.43 | 12.89 | 15.82 | 16.89 | 16.53 | 15.07 | 13.37 | 15.86 | 14.55 | 15.65 | 14.55 | 13.80 | 14.10 | 16.74 | 13.28 | 14.81 |
| 24. *Cyrtodactylus* sp. 3 ITBCZ3002 | **21.41** | 18.11 | 17.35 | 17.20 | 9.29 | 4.87 | 17.35 | 15.83 | 17.50 | 19.57 | 16.59 | 18.49 | 17.43 | 16.90 | 12.63 | 16.74 | 16.90 |
| 25. *Cyrtodactylus* cf. sp. 4 IEBR R.4990 | 16.73 | 12.79 | 13.70 | 14.76 | 14.46 | 14.61 | 14.31 | 7.00 | 14.46 | 14.71 | 14.76 | 14.16 | 13.60 | 14.31 | 14.61 | 14.46 | 13.55 |
| 26. *Cyrtodactylus.* sp. 5 KIZ013699 | 18.55 | 15.27 | 14.20 | 15.46 | 16.38 | 15.85 | 15.28 | 7.62 | 14.60 | 16.36 | 15.86 | 14.91 | 15.64 | 15.07 | 16.04 | 14.59 | 12.51 |
| 27. *Cyrtodactylus* sp. 6 VNMN3372 | 18.00 | 15.25 | 15.79 | 14.15 | 16.53 | 15.63 | 14.33 | 12.83 | 12.36 | 16.18 | 16.92 | 15.46 | 15.24 | 12.82 | 15.84 | 15.81 | 13.91 |
| 28. *C. takouensis* ITBCZ2527 | 17.64 | 13.26 | 11.98 | 13.45 | 16.55 | 16.34 | 12.32 | 12.83 | 15.31 | 13.64 | 13.49 | 12.36 | 13.42 | 12.84 | 15.65 | 12.74 | 11.78 |
| 29. *C. taynguyenensis* ROM32119 | 17.64 | 16.28 | 17.34 | 15.38 | 17.52 | 17.20 | 18.53 | 13.43 | 15.22 | 18.00 | 17.50 | 18.00 | 16.46 | 16.18 | 17.73 | 17.56 | 16.68 |
| 30. *C. thuongae* IEBR A.2013.23 | 17.83 | 14.61 | 14.61 | 16.44 | 17.81 | 17.50 | 16.44 | 14.92 | 15.53 | **0.74** | 17.05 | 3.83 | 14.52 | 14.92 | 16.74 | 13.85 | 15.22 |
| 31. *C. yangbayensis* ITBCZ3540 | 18.90 | 12.79 | 12.33 | 15.22 | 16.90 | 14.76 | 7.92 | 16.13 | 15.22 | 15.22 | 16.13 | 14.31 | 12.83 | 14.31 | 15.98 | 12.79 | 8.83 |
| 32. *Cyrtodactylus* cf. *ziegleri* VNMN2014 | 18.55 | 15.27 | 7.80 | 14.72 | 16.89 | 15.99 | 14.69 | 14.10 | 14.94 | 15.82 | 16.38 | 15.64 | 16.35 | 14.11 | 16.53 | 6.18 | 13.76 |
| 33*. C. ziegleri* ZMMU R-13116-3 | 18.68 | 14.41 | 7.36 | 14.41 | 17.38 | 17.24 | 15.34 | 15.05 | 15.17 | 15.42 | 15.49 | 15.07 | 15.51 | 14.41 | 15.64 | 5.31 | 14.56 |

**Supplementary Table 3.** Uncorrected (“p”) distance matrix showing percentage genetic divergence (COI) (highlighted in bold are the lowest and highest percentage) between species in the *Cyrtodactylus irregularis* group (continued).

|  | 18 | 19 | 20 | 21 | 22 | 23 | 24 | 25 | 26 | 27 | 28 | 29 | 30 | 31 | 32 | 33 |
| --- | --- | --- | --- | --- | --- | --- | --- | --- | --- | --- | --- | --- | --- | --- | --- | --- |
| 18. *C. phuocbinhensis* KH-Res041 | - |  |  |  |  |  |  |  |  |  |  |  |  |  |  |  |
| 19. *C. pseudoquadrivirgatus* ITBCZ3001 | 13.25 | - |  |  |  |  |  |  |  |  |  |  |  |  |  |  |
| 20. *C. pseudoquadrivirgatus* ZMMU R130952 | 13.16 | 8.22 | - |  |  |  |  |  |  |  |  |  |  |  |  |  |
| 21. *C.* cf. *pseudoquadrivirgatus* ITBCZ2532 | 14.99 | 14.55 | 13.27 | - |  |  |  |  |  |  |  |  |  |  |  |  |
| 22. *C. sangi* IEBR R.4988 | 11.72 | 13.93 | 14.96 | 15.86 | - |  |  |  |  |  |  |  |  |  |  |  |
| 23*. Cyrtodactylus* cf. sp.2 ITBCZ1502 | 13.81 | 12.91 | 13.08 | 15.46 | 17.97 | - |  |  |  |  |  |  |  |  |  |  |
| 24. *Cyrtodactylus* sp.3 ITBCZ3002 | 14.16 | 15.94 | 15.81 | 18.23 | 13.09 | 17.60 | - |  |  |  |  |  |  |  |  |  |
| 25. *Cyrtodactylus* cf. sp.4 IEBR R.4990 | 12.79 | 3.07 | 8.94 | 14.81 | 12.94 | 14.36 | 16.13 | - |  |  |  |  |  |  |  |  |
| 26. *Cyrtodactylus.* sp.5 KIZ013699 | 12.75 | 6.36 | 10.43 | 16.55 | 14.34 | 15.27 | 17.63 | 7.30 | - |  |  |  |  |  |  |  |
| 27. *Cyrtodactylus* sp.6 VNMN3372 | 13.64 | 11.64 | 12.02 | 12.73 | 15.97 | 13.64 | 15.78 | 12.18 | 13.27 | - |  |  |  |  |  |  |
| 28. *C. takouensis* ITBCZ2527 | 13.80 | 13.09 | 12.72 | 14.36 | 14.69 | 14.18 | 17.97 | 12.35 | 13.27 | 12.55 | - |  |  |  |  |  |
| 29. *C. taynguyenensis* ROM32119 | 14.63 | 12.73 | 10.59 | 15.82 | 17.29 | 15.64 | 17.51 | 12.80 | 14.55 | 13.46 | 16.18 | - |  |  |  |  |
| 30. *C. thuongae* IEBR A.2013.23 | 14.76 | 14.16 | 16.24 | 15.72 | 16.90 | 14.35 | 17.96 | 15.22 | 16.23 | 15.80 | 13.46 | 17.33 | - |  |  |  |
| 31. *C. yangbayensis* ITBCZ3540 | 12.79 | 14.30 | 15.55 | 15.88 | 14.92 | 14.33 | 16.29 | 14.31 | 14.17 | 13.24 | 11.60 | 15.69 | 14.46 | - |  |  |
| 32. *Cyrtodactylus* cf. *ziegleri* VNMN2014 | 14.37 | 14.55 | 14.32 | 14.18 | 16.16 | 12.18 | 17.78 | 15.06 | 15.27 | 15.64 | 13.09 | 16.18 | 15.62 | 12.16 | - |  |
| 33*. C. ziegleri* ZMMU R-13116-3 | 14.08 | 15.25 | 15.56 | 14.28 | 15.97 | 13.12 | 17.23 | 15.80 | 16.08 | 16.18 | 12.59 | 15.53 | 15.02 | 13.15 | 2.05 | - |

Notes: The genetic divergences between samples of *C. badenensis* are 0.19 – 0.92%; *C. bidoupimontis* are 0.00%; *C. bugiamapensis* are 0.18 – 0.36%; *C. caovansungi* are 0.00 – 0.18%; *C. cattienensis* are 0.00 – 1.22%; *C. chungi* are 0.00%; *C. cucdongensis* are 0.00%; *C. culaochamensis* are 0.46%; *C. cryptus* are 0.00 – 0.18%; *C. dati* are 0.00%; *C. gialaiensis* are 0.30%; *C. huynhi* are 0.00 – 0.30%; *C. irregularis* are 0.15%; *C. kingsadai* are 0.00 – 0.91%; *C. phnomchiensis* are 0.15%; *C. phumyensis* are 0.30%; *C. phuocbinhensis* are 0.15 – 0.91%; *C. sangi* are 0.00 – 0.22%; *C. takouensis* are 0.18%; *C. taynguyenensis* are 0.00%; *C. thuongae* are 0.15%; *C. yangbayensis* are 0.61 – 1.07%.
