# Supplementary material for: ﻿How many more species are out there? Current taxonomy substantially underestimates the diversity of bent-toed geckos (Gekkonidae, Cyrtodactylus) in Laos and Vietnam
Source: Zookeys. 2022 Apr 26;1097:135–52. doi: 10.3897/zookeys.1097.78127 (PMC9848914; doi:10.3897/zookeys.1097.78127)
Supplement: Supplementary material 4 — Table S4 [file zookeys-1097-135_article-78127__-s004.docx]

**Supplementary Table 4.** Uncorrected (“p”) distance matrix showing percentage genetic divergence (COI) (highlighted in bold are the lowest and highest percentage) between species in the *Cyrtodactylus condorensis* group.

|  | 1 | 2 | 3 | 4 | 5 | 6 | 7 | 8 |
| --- | --- | --- | --- | --- | --- | --- | --- | --- |
| 1. *Cyrtodactylus condorensis* KG2014.8 | - |  |  |  |  |  |  |  |
| 2. *C. condorensis* ITBCZ2605 | 2.44 | - |  |  |  |  |  |  |
| 3. *C. condorensis* KIZ1023 | 0.18 | 2.57 | - |  |  |  |  |  |
| 4. *C. eisenmanae* LSUHC8597 | 16.13 | 15.64 | 15.46 | - |  |  |  |  |
| 5. *C. grismeri* TZ68 | 15.83 | 15.07 | 16.10 | **18.05** | - |  |  |  |
| 6. *C. grismeri* ITBCZ690 | 16.18 | 15.11 | 16 | 17.27 | 0.38 | - |  |  |
| 7. *C. leegrismeri* LSUHC11376 | 6.24 | **5.48** | 6.21 | 16.45 | 16.74 | 16.57 | - |  |
| 8. *C. leegrismeri* LSUHC11410 | 6.39 | 5.63 | 6.39 | 16.61 | 16.90 | 16.75 | 0.15 | - |

Notes: The genetic divergences between samples of *C. condorensis* are 0.00 – 2.89%; *C. eisenmanae* are 0.00%; *C. grismeri* are 0.00 – 0.38%; *C. leegrismeri* are 0.15%.
