# Supplementary material for: ﻿How many more species are out there? Current taxonomy substantially underestimates the diversity of bent-toed geckos (Gekkonidae, Cyrtodactylus) in Laos and Vietnam
Source: Zookeys. 2022 Apr 26;1097:135–52. doi: 10.3897/zookeys.1097.78127 (PMC9848914; doi:10.3897/zookeys.1097.78127)
Supplement: Supplementary material 5 — Table S5 [file zookeys-1097-135_article-78127__-s005.docx]

**Supplementary Table 5.** Uncorrected (“p”) distance matrix showing percentage genetic divergence (COI) (highlighted in bold are the lowest and highest percentage) between species in the *Cyrtodactylus chauquangensis* group.

|  | 1 | 2 | 3 | 4 | 5 | 6 | 7 | 8 | 9 | 10 | 11 | 12 | 13 | 14 | 15 | 16 | 17 |
| --- | --- | --- | --- | --- | --- | --- | --- | --- | --- | --- | --- | --- | --- | --- | --- | --- | --- |
| 1. *Cyrtodactylus bobrovi* IEBR A.2015.30 | - |  |  |  |  |  |  |  |  |  |  |  |  |  |  |  |  |
| 2. *C*. cf. *bichnganae* PAT250 | 17.14 | - |  |  |  |  |  |  |  |  |  |  |  |  |  |  |  |
| 3. *C. cucphuongensis* UNS0406 | 6.85 | 16.81 | - |  |  |  |  |  |  |  |  |  |  |  |  |  |  |
| 4*. C. chauquangensis* NA2016.1 | 9.29 | 15.14 | 7.76 | - |  |  |  |  |  |  |  |  |  |  |  |  |  |
| 5*. C. houaphanensis* IEBR A.2013.109 | 6.24 | 17.30 | 7.15 | 8.83 | - |  |  |  |  |  |  |  |  |  |  |  |  |
| 6*. C. huongsonensis* IEBR A.2011.3A | 15.83 | 14.76 | 14.76 | 14.61 | 15.98 | - |  |  |  |  |  |  |  |  |  |  |  |
| 7. *C. martini* UNS0471 | 15.11 | 15.31 | 14.35 | 13.74 | 15.57 | 15.11 | - |  |  |  |  |  |  |  |  |  |  |
| 8. *C. ngoiensis* LPB62-1 | 12.18 | 15.47 | 13.70 | 11.57 | 13.09 | 14.16 | 14.66 | - |  |  |  |  |  |  |  |  |  |
| 9. *C. otai* IEBR A.2015.26 | **3.81** | 16.80 | 7.15 | 9.29 | 6.24 | 15.37 | 15.88 | 13.09 | - |  |  |  |  |  |  |  |  |
| 10. *C. puhuensis* KIZ11665 | 7.48 | 18.99 | 8.60 | 10.76 | 3.30 | 18.28 | 16.21 | 15.45 | 7.45 | - |  |  |  |  |  |  |  |
| 11. *C. soni* HNUE VL.2015.78 | 16.74 | 14.09 | 15.83 | 14.16 | 16.44 | 5.48 | 15.26 | 14.92 | 16.44 | **19.54** | - |  |  |  |  |  |  |
| 12. *C. sonlaensis* IEBR A.2017.1 | 16.13 | 15.27 | 16.59 | 16.74 | 17.66 | 13.39 | 14.49 | 15.37 | 17.20 | 19.07 | 14.00 | - |  |  |  |  |  |
| 13. *Cyrtodactylus* sp.7 KIZ201103 | 17.07 | 15.88 | 15.64 | 14.87 | 16.53 | 16.00 | 3.61 | 14.43 | 17.76 | 17.82 | 16.37 | 15.34 | - |  |  |  |  |
| 14. *C. spelaeus* ZMMU R-13980-3 | 10.01 | 16.99 | 12.28 | 11.79 | 10.99 | 16.93 | 15.07 | 13.54 | 11.31 | 11.67 | 16.12 | 15.62 | 15.40 | - |  |  |  |
| 15. *C. taybacensis* TBU09 | 15.68 | 5.49 | 14.92 | 13.85 | 15.98 | 14.76 | 14.35 | 15.07 | 16.29 | 17.61 | 14.16 | 15.68 | 14.36 | 15.14 | - |  |  |
| 16. *C. vilaphongi* IEBR A.2013.103 | 9.29 | 16.50 | 9.44 | 8.22 | 8.22 | 15.22 | 14.65 | 12.94 | 9.44 | 10.44 | 15.68 | 17.05 | 15.49 | 11.96 | 15.37 | - |  |
| 17. *C. wayakonei* ZFMK91016 | 16.74 | 14.82 | 16.59 | 15.07 | 17.05 | 16.59 | 6.86 | 15.22 | 18.11 | 18.43 | 17.20 | 15.68 | 6.9 | 16.48 | 15.07 | 15.83 | - |

Notes: The genetic divergences between samples of *C. bobrovi* are 0.00 – 0.30%; *C.* cf. *bichnganae* are 0.00 – 3.20%; *C. cucphuongensis* are 0.16 – 0.76%; *C. huongsonensis* are 0.00 – 0.46%; *C. ngoiensis* are 0.00%; *C. otai* are 0.00%; *C. puhuensis* are 0.00 – 2.58%; *C. soni* are 0.00 – 1.22%; *C. sonlaensis* are 0.15%; *C. spelaeus* are 0.66%; *C. taybacensis* are 0.76%; *C. vilaphongi* are 0.00%; *C. wayakonei* are 0.82%.
