# Supplementary material for: ﻿How many more species are out there? Current taxonomy substantially underestimates the diversity of bent-toed geckos (Gekkonidae, Cyrtodactylus) in Laos and Vietnam
Source: Zookeys. 2022 Apr 26;1097:135–52. doi: 10.3897/zookeys.1097.78127 (PMC9848914; doi:10.3897/zookeys.1097.78127)
Supplement: Supplementary material 6 — Table S6 [file zookeys-1097-135_article-78127__-s006.docx]

**Supplementary Table 6.** Uncorrected (“p”) distance matrix showing percentage genetic divergence (COI) (highlighted in bold are the lowest and highest percentage) between species in the *C. brevipalmatus* and *C. intermedius* group.

|  | 1 | 2 | 3 | 4 | 5 | 6 | 7 | 8 |
| --- | --- | --- | --- | --- | --- | --- | --- | --- |
| 1. *Cyrtodactylus hontreensis* KG2014.76 | - |  |  |  |  |  |  |  |
| 2. *C. hontreensis* KG2014.77 | 0.46 | - |  |  |  |  |  |  |
| 3. *C. interdigitalis* LSUHC 11006 | 19.48 | 19.64 | - |  |  |  |  |  |
| 4. *C. intermedius* TZ71 | 18.57 | 18.57 | 19.33 | - |  |  |  |  |
| 5. *C. intermedius* ITBCZ609 | 18.70 | 18.71 | 20.22 | 1.66 | - |  |  |  |
| 6. *Cyrtodactylus* sp.8 PT.2017.215 | 20.40 | 20.55 | **3.81** | 20.40 | 21.74 | - |  |  |
| 7. *Cyrtodactylus* sp.8 VNUF R.2014.50 | 19.64 | 19.79 | **4.41** | 20.24 | 21.55 | **2.13** | - |  |
| 8. *C. phuquocensis* PQ.2015.15 | 16.29 | 16.44 | 20.09 | 9.74 | 9.12 | **21.01** | 20.55 | - |

Notes: The genetic divergences between samples of *C. hontrensis* are 0.46%; *C. intermedius* are 1.66%.
